# Cost-effectiveness of Prefusion F Protein-based Vaccines Against Respiratory Syncytial Virus Disease for Older Adults in the United States

**DOI:** 10.1093/cid/ciad658

**Published:** 2023-11-30

**Authors:** Seyed M Moghadas, Affan Shoukat, Carolyn E Bawden, Joanne M Langley, Burton H Singer, Meagan C Fitzpatrick, Alison P Galvani

**Affiliations:** Agent-Based Modelling Laboratory, York University, Toronto, Ontario, Canada; Agent-Based Modelling Laboratory, York University, Toronto, Ontario, Canada; Department of Microbiology and Immunology, McGill University, Montreal, Quebec, Canada; Canadian Center for Vaccinology, IWK Health Centre and Nova Scotia Health Authority, Dalhousie University, Halifax, Nova Scotia, Canada; Emerging Pathogens Institute, University of Florida, Gainesville, Florida, USA; Center for Vaccine Development and Global Health, University of Maryland School of Medicine, Baltimore, Maryland, USA; Center for Infectious Disease Modeling and Analysis, Yale School of Public Health, New Haven, Connecticut, USA; Center for Infectious Disease Modeling and Analysis, Yale School of Public Health, New Haven, Connecticut, USA

**Keywords:** RSV, older adults, vaccination, simulation, cost-effectiveness

## Abstract

**Background:**

Two prefusion F protein-based vaccines, Arexvy and Abrysvo, have been authorized by the US Food and Drug Administration for protecting older adults against respiratory syncytial virus (RSV)-associated lower respiratory tract illness. We evaluated the health benefits and cost-effectiveness of these vaccines.

**Methods:**

We developed a discrete-event simulation model, parameterized with the burden of RSV disease including outpatient care, hospitalization, and death for adults aged 60 years or older in the United States. Taking into account the costs associated with these RSV-related outcomes, we calculated the net monetary benefit using quality-adjusted life-year (QALY) gained as a measure of effectiveness and determined the range of price-per-dose (PPD) for Arexvy and Abrysvo vaccination programs to be cost-effective from a societal perspective.

**Results:**

Using a willingness-to-pay of $95 000 per QALY gained, we found that vaccination programs could be cost-effective for a PPD up to $127 with Arexvy and $118 with Abrysvo over the first RSV season. Achieving an influenza-like vaccination coverage of 66% for the population of older adults in the United States, the budget impact of these programs at the maximum PPD ranged from $6.48 to $6.78 billion. If the benefits of vaccination extend to a second RSV season as reported in clinical trials, we estimated a maximum PPD of $235 for Arexvy and $245 for Abrysvo, with 2-year budget impacts of $11.78 and $12.25 billion, respectively.

**Conclusions:**

Vaccination of older adults would provide substantial direct health benefits by reducing outcomes associated with RSV-related illness in this population.

## Teaser

Vaccination against RSV-associated lower respiratory tract disease could be cost-effective and substantially reduce illness, hospitalization, and loss of productivity among older adults. Decisions regarding the provision of RSV vaccines to older adults are critical to the real-world impact of vaccination.

## Graphical Abstract

**Graphical Abstract:**
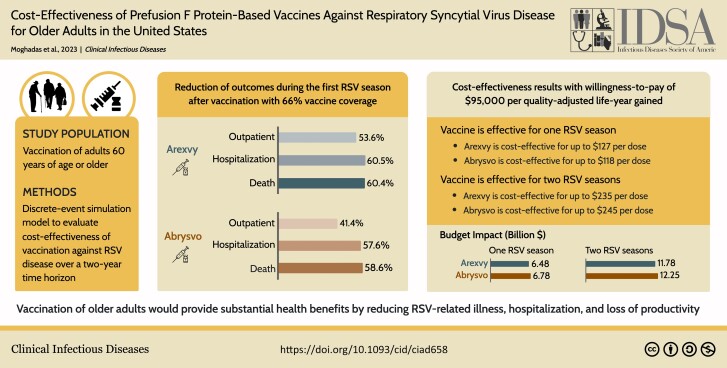
This graphical abstract is also available at Tidbit: https://tidbitapp.io/tidbits/cost-effectiveness-of-prefusion-f-protein-based-vaccines-against-respiratory-syncytial-virus-disease-forolder-adults-in-the-united-states

Respiratory syncytial virus (RSV) is a major cause of lower respiratory tract disease (LRTD) among older adults [1–3], with significant health and socioeconomic burden on a global scale [4]. RSV-related hospitalizations and healthcare costs are exacerbated by the presence of comorbidities, the rates of which have been increasing among older adults [1]. In the United States alone, the annual direct and indirect costs of RSV disease in adults aged 60 years or older are estimated to exceed $3.9 billion [5]. To reduce the burden of RSV disease among older adults, 2 highly efficacious prefusion F protein vaccines (Arexvy and Abrysvo) [6, 7] have been developed, authorized by the US Food and Drug Administration, and recommended by the Centers for Disease Control and Prevention [8, 9].

With the availability of these vaccines, determining vaccination strategies that are cost-effective remains an important component of program implementation. In this study, we conducted a cost-effectiveness analysis of various RSV vaccination programs by developing a discrete-event probabilistic model of RSV outcomes for adults aged 60 years or older in the United States. The model includes important characteristics of the study population with estimates of disease burden and the associated costs. Using stochastic simulations, we calculated the net-monetary benefit (NMB), the incremental cost-effectiveness ratio (ICER), and the budget impact associated with programs evaluated. In addition, we determined the range of vaccine price-per-dose (PPD) within which a program would be cost-effective, while accounting for the reported efficacy estimates of Arexvy and Abrysvo against RSV LRTD. Considering direct and indirect costs of RSV disease outcomes and management, we performed our analysis from a societal perspective.

## METHODS

### Model Structure and Study Population

**Figure 1.**
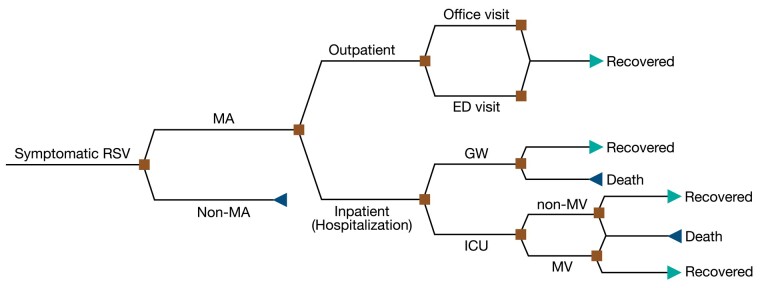
Structure of the discrete-event simulation model applied to scenarios in the abence and presence of interventions for different outcomes. Abbreviations: ED, emergency department; GW, general ward; ICU, intensive care unit; MA, medically attended; MV, mechanical ventilation; RSV, respiratory syncytial virus.

We developed a discrete-event simulation model (Figure 1) with a population of 100 000 adults stratified into age groups of 60 to 64, 65 to 69, 70 to 74, 75 to 79, 80 to 84, and 85 years or older reflecting US demographics [10]. We considered the prevalence of comorbidities across these age groups (Supplementary Table 1) [11], which was used in determining the severe outcomes of RSV disease for adults with 0, 1–3, and ≥4 comorbidities [12].

### RSV-related Outcomes

The model was parameterized with sampled annual incidence of medically attended (MA) RSV cases per 100 000 adults aged 60 years or older, stratified as outpatient (ie, office visits and emergency department [ED] visits) and inpatient (ie, hospitalization in the general ward, and intensive care unit [ICU] admissions) [13]. We uniformly sampled the annual incidence of outpatient office visits from the range 1595–2,669, with a mean of 2133. Annual ED visits were uniformly sampled from the range 23–387, with a mean of 201. The annual incidence of hospitalizations, including ICU admissions, was sampled uniformly from the range 178–250, with a mean of 214. Distribution of hospitalizations among age groups were parameterized based on the average rates reported by RSV-NET for 4 seasons from 2016–17 to 2019–20 [14]. The proportions of hospitalized cases were 6.2%, 12.6%, 26.5%, and 54.8% among age groups 60–64, 65–74, 75–84, and 85 years or older, respectively. Among hospitalized patients, 5.5% had no comorbidities, 78.2% were with 1–3 comorbidities, and 16.3% had ≥4 comorbidities [12]. The rates of ICU admission were set to 24%, 15%, and 12% for patients with 0, 1–3, and ≥4 comorbidities, respectively [12]. Among patients admitted to the ICU, 16.6% required the use of mechanical ventilation (MV) [14]. The death rate for hospitalized patients was sampled uniformly in the range 6.6%–11.0% [15, 16], distributed as 37% from the general ward and 63% from those admitted to ICU [12]. The durations of RSV-related outcomes were sampled for each RSV case from their respective ranges and distributions (Table 1).

**Table 1.** Model Parameters for the Duration of RSV-related Outcomes and Associated Costs

RSV-related Outcome	Duration	Unit	Source
Symptomatic disease, non-MA RSV cases	2–8	d	[17]
Symptomatic disease, MA RSV outpatient care	7–14	d	[15]
Median time interval between onset of symptoms and hospital admission	4	d	[18, 19]
Length of hospitalization in GW	Mean: 6.2 Gamma (1.2258, 5.0582)	d	[20]
Length of hospitalization in ICU	Mean: 4.5 Gamma (1.5625, 2.8802)	d	[21]
Length of stay in GW before ICU admission	1	d	[22]
Length of stay in GW post-ICU	2	d	[23]

RSV-related outcome	Cost estimates	Unit	Source
Office visit	$126	Per visit	[15]
ED visit	$649	Per visit	[12]
Hospitalization, GW	$1116	Per d	[12, 15, 24, 25]
ICU without MV	$3348	Per d	…
ICU with MV	$4870	Per d	…

Abbreviations: GW, general ward; ICU, intensive care unit; MA, medically attended; MV, mechanical ventilation; RSV, respiratory syncytial virus.

### Costs of RSV-related Outcomes

Direct costs of RSV-related outcomes included office visits, ED visits, and hospitalization (Table 1). For indirect costs, productivity loss was calculated for the duration of RSV disease and associated outcomes, as well as the remaining productivity life expectancy [26] for those patients who died (Supplementary Table 2). We considered estimates for annual market and non-market productivity [27] and used the age-stratified proportion of the study population participating in the labor force. To calculate the total productivity loss in the event of death due to RSV, we assumed an annual productivity growth rate of 1% and a discounting rate of 3% [27]. This calculation does not include future unrelated healthcare costs due to cancer, cardiovascular diseases, or other health conditions. For working adults, we accounted for both market and non-market productivity losses. For those out of the labor force, only non-market productivity losses were considered. All costs were converted and inflated to 2023 US dollars.

### RSV Vaccination Strategies and Associated Costs

We considered two scenarios of RSV vaccination coverage of older adults. In the first scenario (S1), we assumed a coverage of 66% similar to the average influenza vaccination coverage from 2010–11 to 2020–21 seasons in the United States for adults aged 65 years or older [28]. For comparison purposes, we also considered a second scenario (S2) with a 100% vaccination coverage for this population. Based on the seasonality of RSV (Supplementary Figure 1) [29], we assumed vaccination begins in September (similar to timelines for influenza vaccination), achieving the target coverage within 2 months in each scenario.

For this analysis, we varied the purchasing cost of a single dose of RSV vaccines between $50 and $500 to determine the price range within which an immunization program would be cost-effective. Based on administration costs of seasonal influenza vaccination, the average cost for administering vaccine was set to $25 per dose [30].

### Efficacy of RSV Vaccines

We considered 2 RSV prefusion F protein vaccines authorized in the United States, Arexvy from GlaxoSmithKline and Abrysvo from Pfizer. The efficacy of a single dose of Arexvy against MA RSV-related LRTD, applied against outpatient care in this study, was estimated at 82.6% (95% confidence interval [CI]: 57.9%–94.1%) over a median follow-up period of 6.7 months [6, 31]. Arexvy efficacy against severe RSV-related LRTD, applied against hospitalization, is estimated at 94.1% (95% CI: 62.4%–99.9%) [6, 31]. Similarly, the efficacy of Abrysvo against MA RSV-related LRTD, applied against outpatient care, is estimated at 65.1% (95.0%% CI: 35.9%–82.0%) through the end of the first RSV season [7, 32]. Abrysvo efficacy against severe RSV-related LRTD, applied against hospitalization in this study, is estimated at 88.9% (95.0% CI: 53.6%–98.7%) [7, 32].

To account for waning immunity, we considered two profiles of temporal decay for vaccine efficacy corresponding to sigmoidal and linear. For the first profile, we fitted a sigmoidal function over a 24-month period to derive point estimates with the same mean efficacy as estimated in clinical trials (Supplementary Figure 2). For Arexvy, we considered a sigmoidal decay over 24 months post vaccination and used an 18-month follow up period with estimates of 67.2% (95% CI: 48.2%–80.0%) against outpatient care and 78.8% (95% CI: 52.6%–92.0%) against hospitalization, derived in the secondary endpoint analysis [6]. Similarly, for Abrysvo we fitted a sigmoidal function over 24 months post vaccination, and used secondary estimates of 48.9% (95.0% CI: 13.7%–70.5%) against outpatient care and 78.6% (95.0% CI: 23.2%–96.1%) against hospitalization during 18 months follow-up [7]. For the linear profile, we used efficacy estimates as reported in clinical trials over the follow-up periods, with a linear decline beginning at 18 months post-vaccination (Supplementary Figure  *A*2).

### Adverse Events of Vaccination

We considered the most frequently solicited systemic adverse reactions (AR) for Arexvy (49.4%) [33] and Abrysvo (27.4%) [34] within 4 to 7 days post-vaccination. We assumed a duration of 1.5 days for any AR [33, 34], with a decrease in the utility values (described below) similar to non-MA RSV cases for the duration of the AR.

### Cost-effectiveness Analysis

To conduct a cost-effectiveness analysis, we calculated the net monetary benefit as NMB=ΔE×WTP−ΔC, where ΔE represents the quality-adjusted life-year (QALY) saved using vaccination compared to no intervention, ΔC is the incremental costs associated with the vaccination scenario, and WTP is the willingness-to-pay threshold for a QALY gain. A vaccination scenario was considered cost-effective if it resulted in a positive NMB. In our main analysis, we calculated the monetary value of health gained using a WTP threshold of $95 000 per QALY [35]. As sensitivity analyses (Supplementary Figures 18–29, Supplementary Tables 11–14), we estimated the NMB and conducted the cost-effectiveness analysis using WTP thresholds of $80 000 and $120 000 [35]. We also estimated the ICER for each vaccination scenario as ΔC/ΔE to measure the additional costs incurred for gaining one QALY. Total QALYs in each scenario were calculated based on the health utility values related to RSV disease and outcomes among different age groups in the study population (Supplementary Table 3) [15, 26, 36]. To account for uncertainty, we sampled utility values from Beta distributions for each RSV case individually and applied the weights associated with RSV-related outcomes (Supplementary Table 3, Supplementary Figure 3), while adjusting for the duration of illness. Cost-effectiveness analyses were conducted from a societal perspective considering both direct and indirect costs. All costs and outcomes were discounted at an annual rate of 3%.

For the primary analysis, we considered a time horizon of a single (first) RSV season post vaccination and derived the number needed to vaccinate to avert one outcome (Supplementary Table 6). In the secondary analysis, the time horizon was set to two RSV seasons in light of the efficacy estimates for a single dose over a 24-month period (Supplementary Figures 18–29, Supplementary Tables 9, 11–14).

The cost-effectiveness analysis was conducted for both a single season and 2 seasons, comparing vaccination scenarios S1 and S2 to the scenario with no intervention. For each scenario of vaccination, we considered three cases for the use of vaccines: (i) Arexvy alone, (ii) Abrysvo alone, and (iii) a combination of Arexvy and Abrysvo with a probability of 50% receiving one of these vaccines to achieve the target coverage. For case (iii), we assumed that the PPD would be the same for both vaccines.

### Ethics and Guidelines

This study used published estimates and publicly available data sources, and thus no ethics approval was required. Consolidated Health Economic Evaluation Reporting Standards (CHEERS) for reporting were followed [37].

## RESULTS

### Health Outcomes

**Figure 2.**
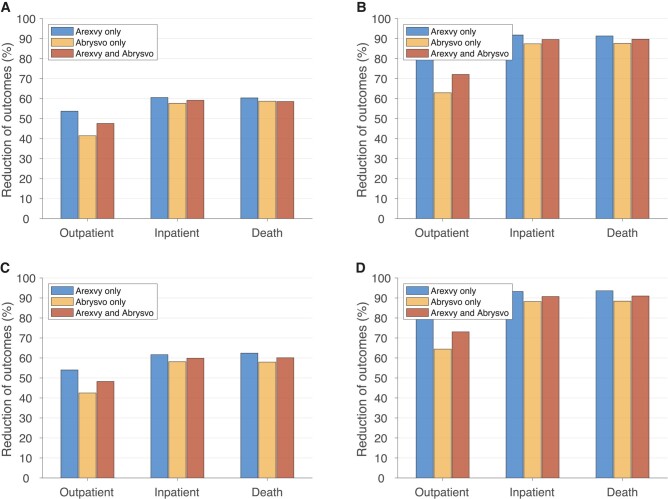
Overall reduction of RSV-related outpatient care (office and ED visits), inpatient care (hospitalization), and death among adults 60 y of age or older, compared to the scenario without vaccination over the first RSV season, with sigmoidal (*A*, *B*) and linear (*C*, *D*) vaccine efficacy profiles. Vaccination coverage was set to 66% (*A*, *C*) and 100% (*B*, *D*). Abbreviations: ED, emergency department; RSV, respiratory syncytial virus.

Using the sigmoidal vaccine efficacy profile (Supplementary Figure 2), S1 with 66% vaccination coverage resulted in a mean reduction of 53.6%, 41.4%, and 47.6% in outpatient care using Arexvy only, Abrysvo only, and a combination of Arexvy and Abrysvo, respectively, for the first RSV season (Figure 2*A*). The corresponding reductions in hospitalizations were 60.5%, 57.6%, and 59.2%. RSV-related deaths were reduced by 60.4%, 58.6%, and 58.5%. Increasing vaccination coverage to 100%, S2 resulted in mean reductions of 81.2%, 62.9%, and 72.1% in outpatient care; 91.7%, 87.4%, and 89.6% in hospitalizations; and 91.3%, 87.6%, and 89.7% in deaths using Arexvy only, Abrysvo only, and a combination of Arexvy and Abrysvo, respectively (Figure 2*B*). When linear vaccine efficacy profiles were used (Supplementary Figure 2), we found an insignificant change in the reduction of outcomes compared to the sigmoidal vaccine efficacy profiles over the first RSV season (Figure 2*C* and 2*D*). The age-specific reduction of outcomes were similar to the overall reduction in the corresponding scenarios of S1 and S2 (Supplementary Figures 4 and 5). Net savings achieved through reduction of RSV-related outcomes and averted loss of productivity were estimated in different age groups (Supplementary Tables 4, 5, 7, and 8).

### Cost-effectiveness of Vaccination Scenarios

Under S1 with 66% vaccination coverage and sigmoidal vaccine efficacy profiles, Arexvy resulted in 59.19 (95% CI: 58.25–60.14) QALY gain over the first RSV season (Table 2). A program with Abrysvo saved 56.71 (95% CI: 55.82 57.67) QALYs. Combination of Arexvy and Abrysvo resulted in 57.74 (95% CI: 56.78–58.72) QALY gains. Increasing vaccination coverage to 100% in S2 resulted in QALY gains of 89.06 (95% CI: 88.09–90.09), 84.01 (95% CI: 82.87–85.10), and 87.27 (95% CI: 86.23–88.31) using Arexvy only, Abrysvo only, and combination of both vaccines, respectively. Similar estimates of QALY gains were obtained for vaccination programs using the linear vaccine efficacy profiles (Table 2).

**Table 2.** Model Estimates of Cost-effectiveness Analyses for Vaccination Programs With Arexvy Only, Abrysvo Only, and Combination of Arexvy and Abrysvo Over the First RSV Season in a Population of 100 000 Adults Aged 60 y or Older at the WTP of $95 000

Vaccination Scenario	Maximum PPD, $	Incremental Costs, $ (95% CI)	QALYs Saved (95% CI)	ICER (95% CI)	Budget Impact per 100,000, $	National Budget Impact, $ Billion
S1 with sigmoidal vaccine efficacy						
Arexvy only	127	5 562 363 (5 510 302 to 5 614 552)	59.19 (58.25 to 60.14)	93 981 (91 629 to 96 362)	8 699 485	6.78
Abrysvo only	118	5 368 121 (5 321 784 to 5 416 761)	56.71 (55.82 57.67)	94 651 (92 275 to 96 913)	8 209 939	6.48
Arexvy and Abrysvo	122	5 440 866 (5 388 861 to 5 491 542)	57.74 (56.78 to 58.72)	94 234 (91 837 to 96 604)	8 429 527	6.65
S2 with sigmoidal vaccine efficacy						
Arexvy only	126	8 368 448 (8 314 923 to 8 424 116)	89.06 (88.09–90.09)	93 968 (92 339 to 95 595)	13 092 291	10.65
Abrysvo only	115	7 936 899 (7 876 114 to 7 995 439)	84.01 (82.87–85.10)	94 471 (92 607 to 96 409)	12 149 858	9.67
Arexvy and Abrysvo	122	8 290 977 (8 232 919 to 8 348 981)	87.27 (86.23–88.31)	95 004 (93 301 to 96 785)	12 779 360	10.16
S1 with linear vaccine efficacy						
Arexvy only	132	5 771 240 (5 718 678 to 5 821 915)	61.37 (60.45–62.31)	94 035 (91 732 to 96 320)	9 014 880	7.11
Abrysvo only	117	5 300 596 (5 252 638 to 5 349 812)	55.99 (55.06–56.87)	94 664 (92 365 to 97 149)	8 137 178	6.42
Arexvy and Abrysvo	126	5 615 828 (5 563 478 to 5 672 281)	59.21 (58.21–60.15)	94 848 (92 556 to 97 408)	8 680 022	6.84
S2 with linear vaccine efficacy						
Arexvy only	130	8 627 058 (8 571 284 to 8 685 392)	91.80 (90.77–92.78)	93 978 (92 413 to 95 651)	13 461 485	10.62
Abrysvo only	116	7 940 563 (7 880 804 to 7 997 330)	84.56 (83.48–85.55)	93 906 (92 146 to 739)	12 232 195	9.65
Arexvy and Abrysvo	123	8 290 016 (8 234 388 to 8 345 572)	88.12 (87.15–89.10)	94 081 (92 427 to 95 749)	12 842 803	10.13

All strategies were compared to the baseline with no intervention.

Abbreviations: CI confidence interval; ICER, incremental cost-effectiveness ratio; PPD, price per dose; QALY, quality-adjusted life-year; RSV, respiratory syncytial virus; WTP, willingness to pay.

**Figure 3.**
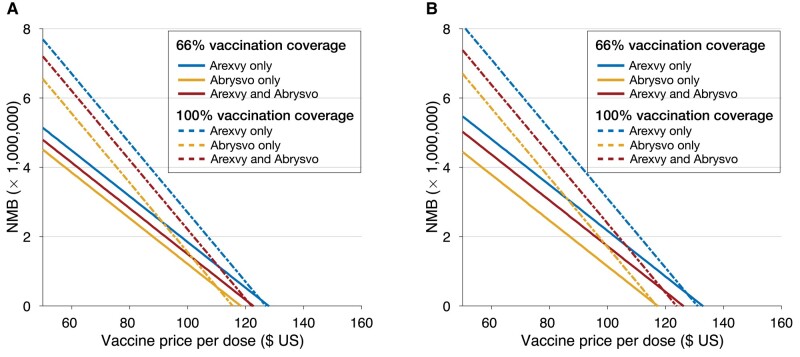
Estimated NMB over the first RSV season as a function of price per dose for Arexvy and Abrysvo with different coverage of vaccination, and with sigmoidal (*A*) and linear (*B*) vaccine efficacy profiles. For scenarios using both Arexvy and Abrysvo, each vaccine was assumed to have 50% of the target coverage with the same price per dose. Abbreviations: NMB, net monetary benefit; RSV, respiratory syncytial virus.

We determined the maximum PPD below which programs with Arexvy, Abrysvo, or combination of both vaccines would be cost-effective (ie, NMB > 0) at a WTP of $95 000 per QALY gained over the course of the first RSV season (Table 2). Using sigmoidal vaccine efficacy profiles (Figure 3*A*), the maximum PPD for a positive NMB was $127 for Arexvy only, $118 for Abrysvo only, and $122 for combination of both vaccines at 66% vaccination coverage, with probabilities of 81%, 61%, and 74% being cost-effective at the strategy-specific PPDs, respectively (Supplementary Figures 6 and 8). With 100% vaccination coverage, the corresponding maximum PPDs were $126, $115, and $122, with cost-effective probabilities of 89%, 70%, and 49%, respectively.

With linear vaccine efficacy profiles, the maximum PPD for a positive NMB was $132 for Arexvy only, $117 for Abrysvo only, and $126 for a combination of both vaccines in S1 (Table 2, Figure 3*B*). At these PPDs, the corresponding probabilities of the vaccination programs being cost-effective were 81%, 62%, and 55%. Under S2, the maximum PPD was $130 for Arexvy, $116 for Abrysvo, and $123 for combination of both vaccines, with cost-effective probabilities of 90%, 90%, and 86%, respectively (Supplementary Figures 7 and 9).

### Budget Impact

With sigmoidal vaccine efficacy profiles, the budget impact to the healthcare system for the first RSV season at the maximum PPD, after discounting for the savings achieved through the reductions of outpatient and inpatient care, was estimated to range from $6.48 to $6.78 billion in S1, and from $9.67 to $10.65 billion in S2 for approximately 79 million older adults in the US (Table 2). The budget impact using linear vaccine efficacy profiles were similar and ranged from $6.42 to $7.11 billion in S1, and from $9.65 to $10.62 billion in S2.

### Secondary Analysis

Our results for a time-horizon of 2 RSV seasons (Supplementary Figures 10–17) indicate that the maximum PPD below which vaccination programs are cost-effective at the WTP of $95 000 per QALY depends on the vaccine efficacy. For example, with a sigmoidal decay of vaccine efficacy, the maximum PPD was $210 for an Arexvy-only program, $197 for an Abrysvo-only program, and $205 when a combination of Arexvy and Abrysvo vaccines were used with 66% vaccination coverage (Supplementary Table 10). However, for the linear vaccine efficacy profiles, the corresponding maximum PPDs for these vaccination programs increased to $235, $245, and $241. Similar PPDs were estimated with 100% vaccination coverage using sigmoidal and linear vaccine efficacy profiles, indicating the sensitivity of PPD to efficacy profiles over two RSV seasons. The budget impact of these programs over two years ranged from $10.02 to $18.61 billion for 79 million adults aged 60 years or older depending on the coverage and vaccine efficacy profiles (Supplementary Table 10).

## DISCUSSION

In this study, we evaluated the cost-effectiveness of 2 recently approved prefusion F protein-based RSV vaccines for older adults. We found that vaccination of adults aged 60 years or older could be cost-effective depending on the price, as well as the durability of vaccine efficacy. Our results indicate that achieving a 66% vaccination coverage akin to influenza season for older adults would substantially alleviate the burden of RSV-related illness. At the national level, this would require a financial commitment of up to $8.18 billion to cover the costs of both purchasing vaccines at $132 per dose and administration to immunize about 52 million older adults. The health benefits estimated in our study are under the assumption that the majority of older adults can afford to access the vaccine. Thus, Medicare, Medicaid, and private insurers decisions regarding the provision of RSV vaccines to older adults are critical to the real-world impact of vaccination.

Although published estimates on efficacy of RSV vaccines are encouraging [6, 7], the real-world effectiveness and durability are still unknown. Such estimates are critically important for decision making on effective and cost-effective programs [38], particularly for subpopulations with elevated risk factors. Given the characteristics of the target population with comorbidities and immunosenescence, the effectiveness and durability of RSV vaccines in a real-world setting is likely to be lower than those reported in clinical trials. We found that the health benefits of vaccination are sensitive to assumptions about vaccine waning, also affecting the cost-effective PPD and anticipated budget impact over a 2-year time horizon. Furthermore, the uncertainty about the benefits of vaccination beyond the first RSV season suggests that the results of cost-effectiveness analysis over a 1-year time-horizon would be more appropriate for informing policy decisions on vaccination campaigns.

Although the model developed in this study is comprehensive in its structure and accounts for parameter uncertainty at the individual level, there are limitations to consider. First, our analysis does not consider the complexity of RSV transmission dynamics. Although no data exist regarding the herd effects of RSV vaccination, it is possible that health benefits could extend beyond just the reduction of disease outcomes by lowering incidence or transmission rates in the population. Second, given the rarity of grade 3 reactions, we did not consider potential short-term costs and loss of productivity associated with treatment of more severe adverse reactions, which may affect estimates of PPD. Third, although we estimated the net savings associated with direct healthcare costs and productivity from vaccinating different age groups in the population study, the estimates of PPD were not stratified by age. Fourth, our model accounted for RSV-related outcomes within the first 2 years post-vaccination. However, longer-term sequelae of RSV infection (eg, wheezing and asthma), as well as potential benefits of vaccination beyond the first 2 years may increase PPD estimates. Finally, our analysis did not account for additional indirect costs incurred as a result of out-of-pocket expenses, or productivity losses due to informal care provided by families of RSV patients.

In conclusion, our study shows that vaccination against RSV-associated LRTD could be cost-effective and reduce the burden of illness substantially among older adults. Additional evidence of vaccine effectiveness at the population level would be required to alleviate uncertainty on longer-term health benefits and cost-effectiveness of vaccination beyond a single RSV season.

## Supplementary Data

Supplementary materials are available at *Clinical Infectious Diseases* online. Consisting of data provided by the authors to benefit the reader, the posted materials are not copyedited and are the sole responsibility of the authors, so questions or comments should be addressed to the corresponding author.

## Supplementary Material

ciad658_Supplementary_Data

## Notes

***Author Contributions*.** S. M. M. and A. P. G. conceived the study; S. M. M. designed the model framework; C. B. and S. M. M. collected input parameters; A. S. developed the computational model and performed simulations; S. M. M. analyzed the simulations data and wrote the first draft of the manuscript; J. M. L., B. H. S., M. C. F., and A. P. G. provided insights into the analysis and interpretation of the results; all authors contributed to the writing.

The computational model is available at: https://github.com/affans/rsv-ce-adults.

***Financial support*.** S. M. M. acknowledges support from the Natural Sciences and Engineering Research Council of Canada Discovery Grant and Alliance Grant (ALLRP 576914—22). M. C. F. acknowledges support from the National Institutes of Health (grant number 5 K01 AI141576). A. P. G. acknowledges support from the National Institutes of Health (grant number R01 AI151176), National Science Foundation (grant number 1918784), and The Notsew Orm Sands Foundation.
