# Supplementary material for: Cost-effectiveness of Prefusion F Protein-based Vaccines Against Respiratory Syncytial Virus Disease for Older Adults in the United States
Source: Clin Infect Dis. 2023 Nov 30;78(5):1328–35. doi: 10.1093/cid/ciad658 (PMC11093660; doi:10.1093/cid/ciad658)
Supplement: ciad658_Supplementary_Data [file ciad658_supplementary_data.docx]

**Supplementary Data**

**Cost-Effectiveness of Prefusion F Protein-Based Vaccines Against Respiratory Syncytial Virus Disease for Older Adults in the United States**

Seyed M. Moghadas,^1^ Affan Shoukat,^1^ Carolyn Bawden,^2^ Joanne M. Langley,^3^ Burton H Singer,^4^ Meagan C. Fitzpatrick,^5,6^ Alison P. Galvani^6^

^1^ Agent-Based Modelling Laboratory, York University, Toronto, Ontario, Canada

^2^ McGill University, Montreal, Quebec, Canada
^3^ Canadian Center for Vaccinology, IWK Health Centre and Nova Scotia Health Authority, Dalhousie University, Halifax, Nova Scotia, Canada

^4^ Emerging Pathogens Institute, University of Florida, Gainesville, Florida, USA

^5^ Center for Vaccine Development and Global Health, University of Maryland School of Medicine, Baltimore, MD, USA

^6^ Center for Infectious Disease Modeling and Analysis, Yale School of Public Health, New Haven, Connecticut, USA

This supplemental provides additional information for parameterization of the model, with tables and figures supporting the results described in the main text for cost-effectiveness analysis of vaccination programs over one and two RSV seasons.

*Model implementation*

The model was simulated stochastically using Monte-Carlo sampling for a total of 1000 independent realizations. In each realization, model parameters (**Table 1 of the Main Text**) were sampled for each individual from their respective distributions or estimated ranges, thus probabilistically accounting for the sensitivity of the outcomes with respect to input values. To generate 95% confidence intervals around point estimates, we employed a nonparametric, bias-corrected and accelerated bootstrap technique with 1000 replicates. The computational model is available at: <https://github.com/affans/rsv-ce-adults>.

**Table A1.** Characteristics of the population age groups.[^1,2^](https://www.zotero.org/google-docs/?kTUpw6)

| **Age group** | **Remaining healthy life** | **Number of comorbidities** | | |
| --- | --- | --- | --- | --- |
|  |  | **0** | **1–3** | **≥4** |
| 60-64 | 14.8 | 32.0% | 58.2% | 9.8% |
| 65-69 | 11.9 | 26.5% | 62.5% | 11.0% |
| 70-74 | 9.2 | 21.5% | 65.0% | 13.5% |
| 75-79 | 6.8 | 17.0% | 67.5% | 15.5% |
| 80-84 | 4.7 | 14.5% | 67.5% | 18.0% |
| 85+ | 3.1 | 11.5% | 66.5% | 22.0% |

**
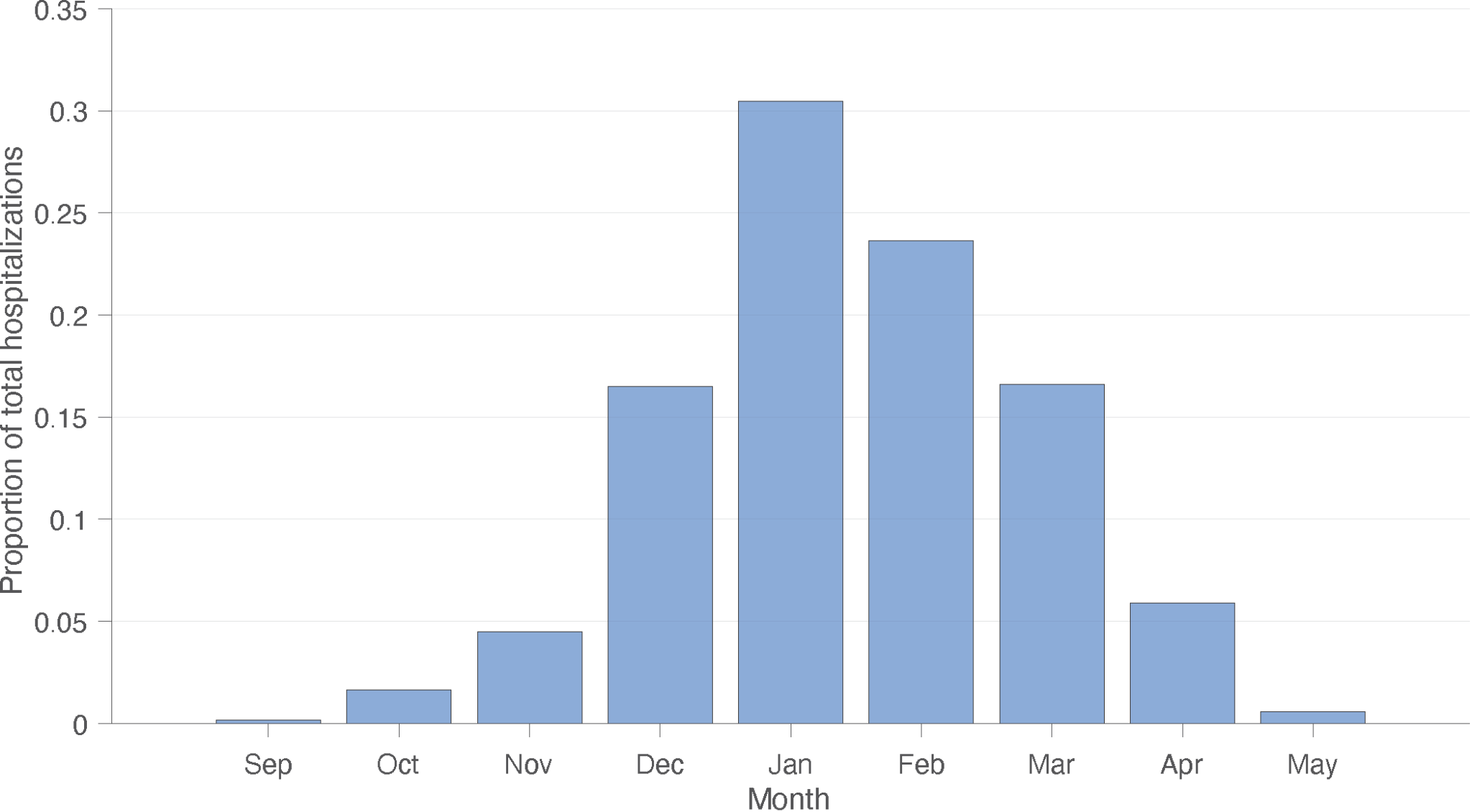
**

**Figure A1.** Seasonality distribution of RSV-related hospitalizations for adults aged 65 years or older, based on an average of 4 seasons from 2016-17 to 2019-2020 reported in the US.[^3^](https://www.zotero.org/google-docs/?EuXKKU) The same distribution was assumed for outpatient care during RSV season.

**Table A2.** Model parameters for calculation of productivity loss due to RSV-related outcomes.[^4,5^](https://www.zotero.org/google-docs/?j16Pz0) We considered a 1% growth productivity per year and a 3% discounting rate on an annual basis.[^5^](https://www.zotero.org/google-docs/?hSOvdl) For working adults, the productivity loss due to death included both market and non-market productivity. For those out of the labor force, we considered only non-market productivity loss due to RSV-related death.

| **Age group** | **Labor force participations** | **Annual market productivity (2023)** | **Annual non-market productivity (2023)** | **productivity years lost due to death** | **Market productivity loss due to death** | **Non-market productivity loss due to death** | **Average QALY loss due to death** |
| --- | --- | --- | --- | --- | --- | --- | --- |
| 60-64 | 63.2% | $46,271 | $25,177 | 15 | $607,221 | $330,401 | 9.47 |
| 65-69 | 25.7% | $24,952 | $26,176 | 12 | $269,429 | $282,645 | 7.79 |
| 70-74 |  | $11,691 | $25,525 | 9 | $97,406 | $212,668 | 5.93 |
| 75-79 | 8.8% | $5,253 | $22,898 | 7 | $34,697 | $151,245 | 4.49 |
| 80-84 |  | $2,540 | $16,772 | 5 | $12,216 | $80,666 | 2.97 |
| 85+ |  | $1,011 | $5,467 | 3 | $2,974 | $16,085 | 1.49 |

The annual market productivity for working adults in 2023 was estimated at $46,271, $24,952, $11,691, $5,253, $2,540, and $1,011 for working adults aged 60-64, 65-69, 70-74, 75-79, 80-84, and 80 years or older, respectively (**Table A2**). These estimates correspond to daily market productivity of $175.3, $94.5, $44.3, $19.9, $9.6, and $3.8 for 22 working days per month among working adults in the corresponding age groups. The annual non-market productivity for these age groups in 2023 were $25,177, $26,176, and $25,525, $22,898, $16,772, and $5,467 with daily non-market productivity of $69.0, $71.7, $69.9, $62.7, $45.9, and $15.0, respectively.

**Temporal decline of vaccine efficacy**

To parameterize the model with temporal efficacy of Arexvy and Abrysvo vaccines, we considered a sigmoidal decay function over a 24-month period, given by

[
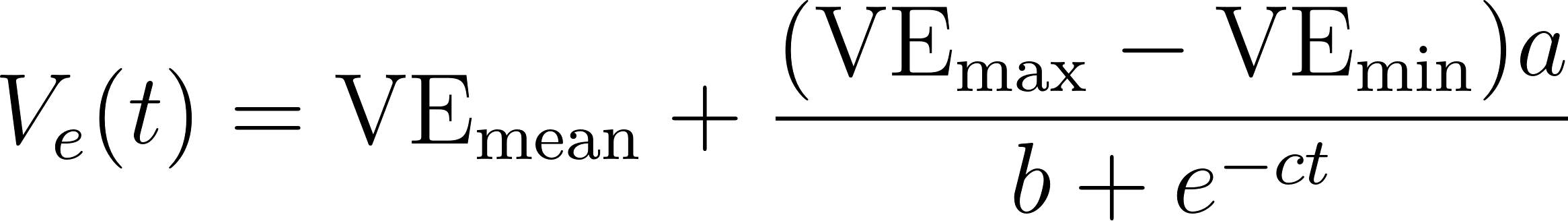
](https://www.codecogs.com/eqnedit.php?latex=V_e(t)%3D%5Ctext%7BVE%7D_%5Ctext%7Bmean%7D%2B%5Cfrac%7B(%5Ctext%7BVE%7D_%5Ctext%7Bmax%7D-%5Ctext%7BVE%7D_%5Ctext%7Bmin%7D)a%7D%7Bb%2Be%5E%7B-ct%7D%7D#0)

where [
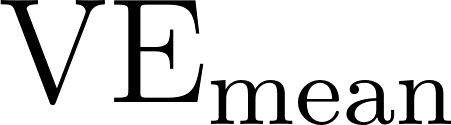
](https://www.codecogs.com/eqnedit.php?latex=%5Ctext%7BVE%7D_%5Ctext%7Bmean%7D#0) is the mean efficacy estimated during the first follow-up period post vaccination, [
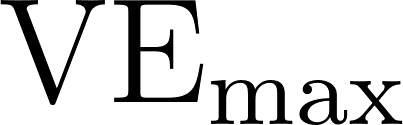
](https://www.codecogs.com/eqnedit.php?latex=%5Ctext%7BVE%7D_%5Ctext%7Bmax%7D#0) and [
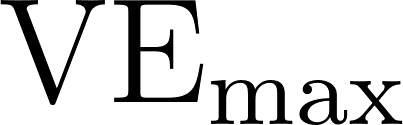
](https://www.codecogs.com/eqnedit.php?latex=%5Ctext%7BVE%7D_%5Ctext%7Bmax%7D#0) are the maximum and minimum efficacy estimates during the entire study period. Assuming that the vaccine efficacy reduced to zero at 24 months after vaccination, we estimated the parameters [
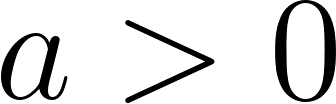
](https://www.codecogs.com/eqnedit.php?latex=a%3E0#0), [
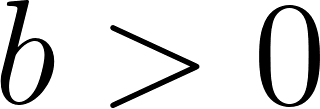
](https://www.codecogs.com/eqnedit.php?latex=b%3E0#0), and [
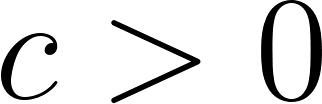
](https://www.codecogs.com/eqnedit.php?latex=c%3E0#0) (using curve fitting function in Matlab) to derive estimates with the same mean efficacy as estimated in clinical trials.[^6,7^](https://www.zotero.org/google-docs/?Y7a6hX) Figure A2 illustrates the decline of protection efficacy of Arexvy and Abrysvo over a 24-month period post-dose for different outcomes.

We also considered linear vaccine efficacy profiles with the mean estimates reported in clinical trials over the follow-up periods for one and two RSV seasons (Figure A2).[^6,7^](https://www.zotero.org/google-docs/?x4ek1E)

**
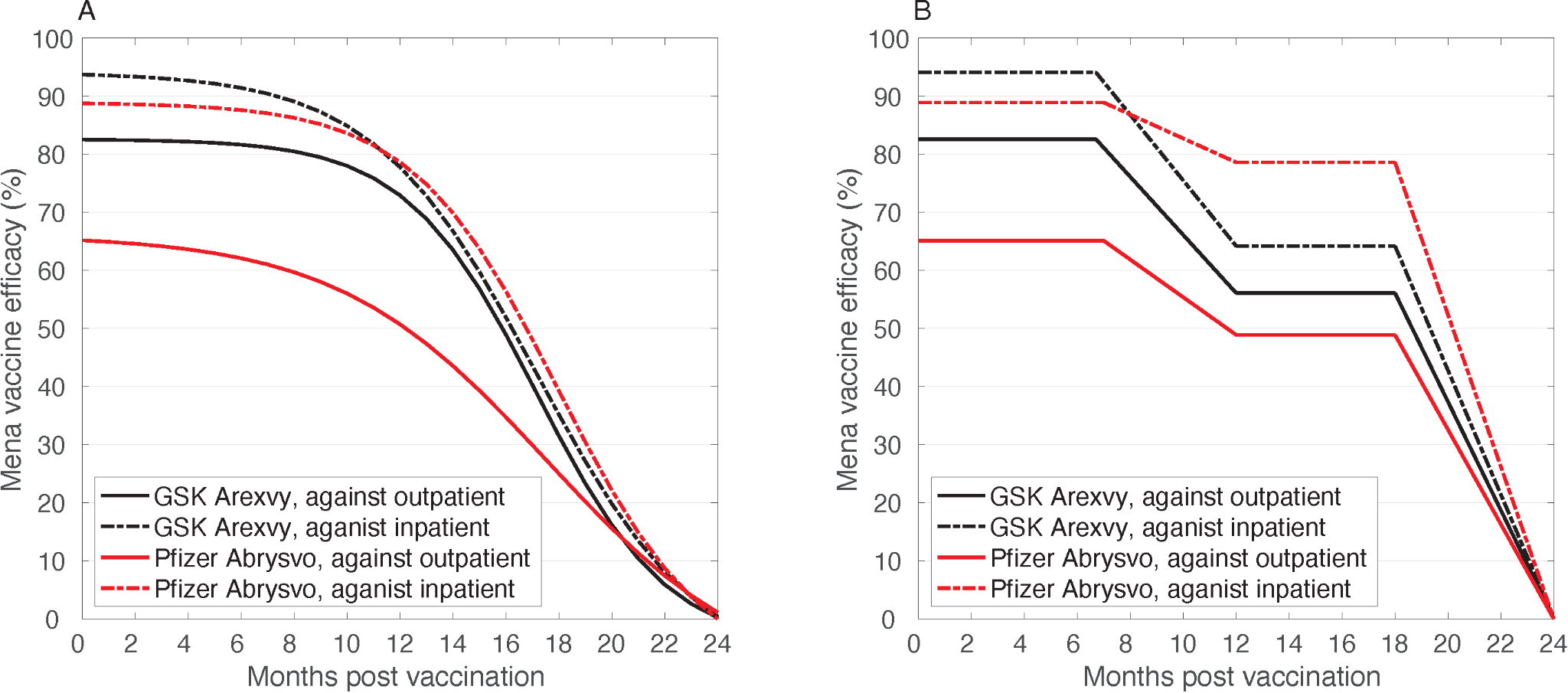
**

**Figure A2.** (A) Temporal decay of efficacy using sigmoidal fit over 24 months after single dose of Arexvy and Abrysvo vaccines against outpatient (office and ED visits) and inpatient care (hospitalization). (B) Efficacy estimates reported in clinical trials for a single dose of Arexvy and Abrysvo vaccines against outpatient (office and ED visits) and inpatient care (hospitalization) over an 18-month follow-up period. After 18 months, the efficacies were assumed to decline linearly to zero.

**Distributions of utilities**

To calculate the utility values during RSV disease, we applied the utility weights associated with outcomes to the sampled utility value for each individual from the distribution of utilities for each age group (Table A3). Weights were 0.76 for outpatient care (office or ED visit), 0.35 for hospitalized non-ICU patients, and 0.1 for hospitalized ICU patients.[^2,8^](https://www.zotero.org/google-docs/?IDMv5c) The utility weight for non-MA RSV cases during the symptomatic illness was set to 0.88, assuming a 50% lower decrement than MA RSV outpatient care. The duration of effect was assumed to be the same as the duration of outcome. Total QALYs were calculated by adding utility values for the duration of outcome and outside the illness duration in each scenario.

**Table A3.** Distribution of utility values used in the cost-effectiveness analysis with average utility values for population age groups.[^2^](https://www.zotero.org/google-docs/?LpQbod)

| **Age group** | **Utility distribution** | **Mean** | **Standard deviation** |
| --- | --- | --- | --- |
| 60-64 | Beta(162.61, 48.57) | 0.77 | 0.029 |
| 65-69 | Beta(141.60, 44.72) | 0.76 | 0.031 |
| 70-74 | Beta(112.16, 39.41) | 0.74 | 0.036 |
| 75-79 | Beta(76.03, 32.58) | 0.70 | 0.044 |
| 80-84 | Beta(41.96, 24.64) | 0.63 | 0.059 |
| 85+ | Beta(33.08, 31.78) | 0.51 | 0.062 |

**
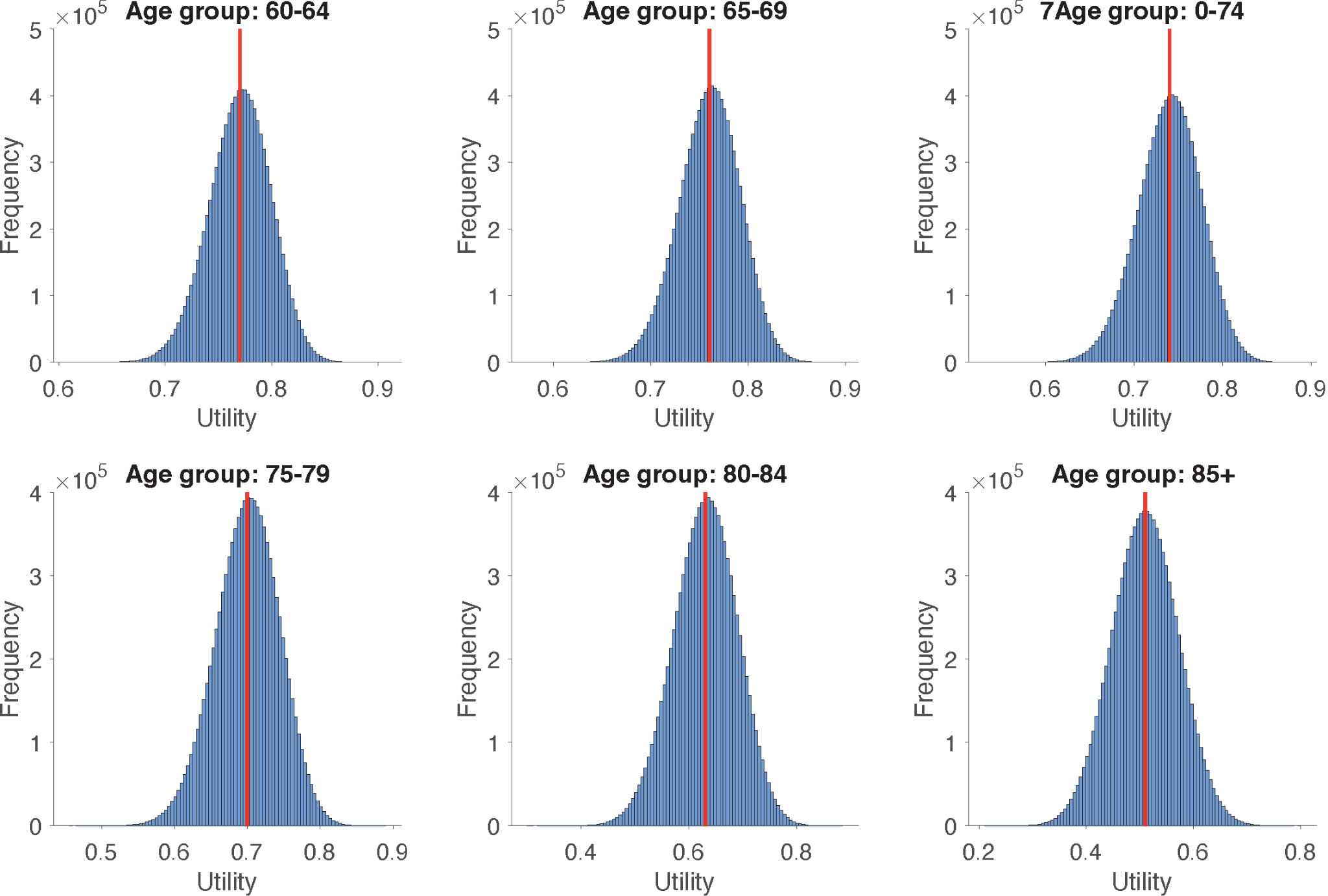
Figure A3.** Distribution of utility values for different age groups without RSV.

**Cost-effectiveness analysis with the WTP of $95,000 per QALY gained during the first RSV season**


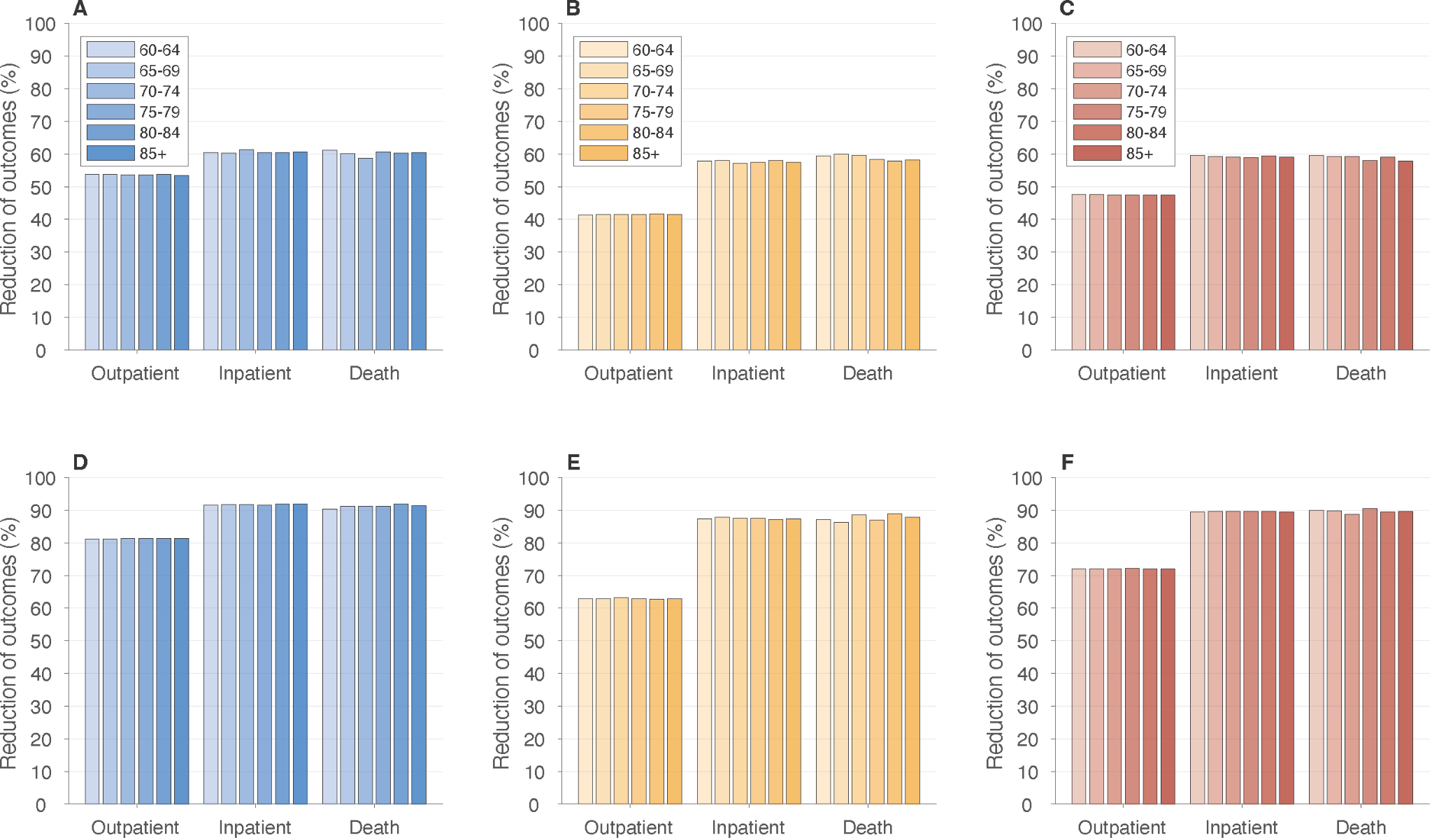
**Figure A4.** Age-specific reductions of outpatient care, hospitalization and deaths achieved in S1 with 66% vaccination coverage (A,B,C) and S2 with 100% vaccination coverage (D,E,F) over a single RSV season. Scenarios correspond to the use of Arexvy vaccine only (A,D); Abrysvo vaccine only (B,E); and a combination of Arexvy and Abrysvo vaccines (C,F), with sigmoidal vaccine efficacy profiles.


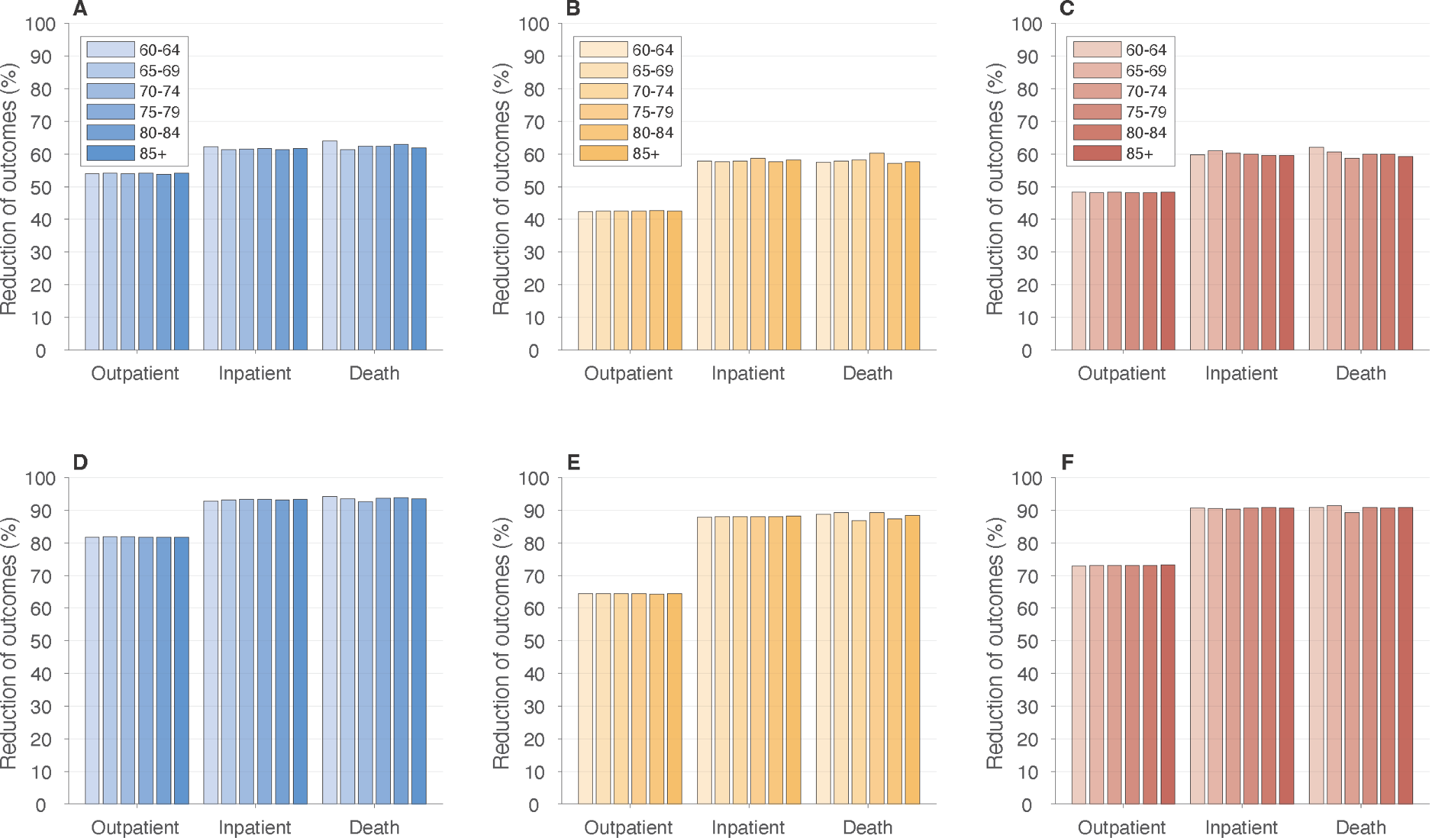
**Figure A5.** Age-specific reductions of outpatient care, hospitalization and deaths achieved in S1 with 66% vaccination coverage (A,B,C) and S2 with 100% vaccination coverage (D,E,F) over a single RSV season. Scenarios correspond to the use of Arexvy vaccine only (A,D); Abrysvo vaccine only (B,E); and a combination of Arexvy and Abrysvo vaccines (C,F), with linear vaccine efficacy profiles.

**Table A4.** Estimated net savings of RSV-related outcomes achieved over the first RSV season using Arexvy in different age groups per 100,000 adults 60 years of age or older.

| **Age group** | **Outpatient** | **Inpatient** | **Productivity**  **(market and non-market)** |
| --- | --- | --- | --- |
| *S1 with sigmoidal vaccine efficacy* | | | |
| 60-64 | $57,080 | $68,939 | $1,412,534 |
| 65-69 | $50,429 | $75,428 | $735,896 |
| 70-74 | $40,737 | $67,918 | $316,923 |
| 75-79 | $27,116 | $180,606 | $395,879 |
| 80-84 | $16,717 | $113,131 | $195,213 |
| 85+ | $8,805 | $625,644 | $80,643 |
| *S2 with sigmoidal vaccine efficacy* | | | |
| 60-64 | $86,136 | $102,190 | $2,132,231 |
| 65-69 | $75,626 | $113,765 | $1,104,040 |
| 70-74 | $61,668 | $100,266 | $479,710 |
| 75-79 | $40,963 | $274,144 | $593,792 |
| 80-84 | $25,239 | $170,184 | $292,942 |
| 85+ | $13,574 | $943,694 | $121,389 |
| *S1 with linear vaccine efficacy* | | | |
| 60-64 | $57,116 | $70,367 | $1,479,223 |
| 65-69 | $49,933 | $76,892 | $753,070 |
| 70-74 | $40,459 | $66,516 | $322,374 |
| 75-79 | $27,098 | $186,448 | $408,264 |
| 80-84 | $16,628 | $114,341 | $198,385 |
| 85+ | $8,723 | $632,745 | $82,180 |
| *S2 with linear vaccine efficacy* | | | |
| 60-64 | $86,292 | $105,693 | $2,154,924 |
| 65-69 | $75,622 | $117,490 | $1,167,045 |
| 70-74 | $61,489 | $102,276 | $491,300 |
| 75-79 | $40,977 | $281,873 | $599,441 |
| 80-84 | $25,108 | $171,947 | $298,432 |
| 85+ | $13,156 | $956,035 | $123,844 |

**Table A5.** Estimated net savings of RSV-related outcomes achieved over the first RSV season using Abrysvo in different age groups per 100,000 adults 60 years of age or older.

| **Age group** | **Outpatient** | **Inpatient** | **Productivity**  **(market and non-market)** |
| --- | --- | --- | --- |
| *S1 with sigmoidal vaccine efficacy* | | | |
| 60-64 | $42,940 | $66,193 | $1,265,097 |
| 65-69 | $37,867 | $73,561 | $688,523 |
| 70-74 | $30,597 | $63,816 | $277,841 |
| 75-79 | $20,072 | $176,836 | $359,933 |
| 80-84 | $12,397 | $107,464 | $176,330 |
| 85+ | $5,048 | $591,121 | $74,243 |
| *S2 with sigmoidal vaccine efficacy* | | | |
| 60-64 | $65,903 | $99,118 | $1,852,841 |
| 65-69 | $57,878 | $111,049 | $1,020,680 |
| 70-74 | $47,177 | $93,353 | $422,755 |
| 75-79 | $30,846 | $260,625 | $535,011 |
| 80-84 | $18,771 | $163,110 | $269,355 |
| 85+ | $7,968 | $894,278 | $112,384 |
| *S1 with linear vaccine efficacy* | | | |
| 60-64 | $44,759 | $64,894 | $1,269,974 |
| 65-69 | $39,328 | $73,022 | $665,563 |
| 70-74 | $31,888 | $62,850 | $285,453 |
| 75-79 | $21,035 | $174,718 | $364,094 |
| 80-84 | $12,777 | $107,537 | $177,098 |
| 85+ | $5,463 | $596,784 | $74,167 |
| *S2 with linear vaccine efficacy* | | | |
| 60-64 | $68,142 | $97,845 | $1,936,615 |
| 65-69 | $59,762 | $110,501 | $1,013,433 |
| 70-74 | $48,450 | $95,215 | $418,032 |
| 75-79 | $31,985 | $265,571 | $545,123 |
| 80-84 | $19,507 | $162,477 | $265,916 |
| 85+ | $8,407 | $899,804 | $112,653 |

*Number needed to vaccinate (NNV)*

The mean number of adults in the study population who needed to be vaccinated to avert one outpatient visit during the first RSV season with sigmoidal vaccine efficacy profiles ranged from 53 to 69 (**Table A6**). Mean estimated NNV to avert one hospitalization ranged from 507 to 534, and from 5,973 to 6,174 to prevent one death. Similar ranges of NNV were found using linear vaccine efficacy profiles (**Table A6**). At the national level with a population of approximately 79 million older adults in the US, these findings indicate that 66% vaccination coverage of adults aged 60 years or older could avert over 982,000 outpatient (office and ED) visits, 102,000 hospitalizations, and 8700 deaths associated with RSV disease over the first RSV season.

**Table A6.** Number of vaccine doses needed to avert one outcome over the first RSV season with temporal vaccine efficacy using sigmoidal fit and linear average estimates (**Figures A2**).

| **Outcome** | **NNV to avert one outcome: mean (95% CI)** | | |
| --- | --- | --- | --- |
|  | **Arexvy only** | **Abrysvo only** | **Arexvy and Abrysvo** |
| *Sigmoidal vaccine efficacy* | | | |
| Outpatient | 53  (52 to 53) | 69  (68 to 69) | 60  (59 to 60) |
| Hospitalization | 507  (504 to 511) | 534  (530 to 537) | 520  (516 to 523) |
| Death | 5,973  (5,882 to 6,068) | 6,174  (6,075 to 6,274) | 6,120  (6,022 to 6,220) |
| *Linear vaccine efficacy* | | | |
| Outpatient | 52  (52 to 53) | 66  (65 to 66) | 59  (58 to 59) |
| Hospitalization | 500  (497 to 504) | 532  (528 to 536) | 514  (510 to 518) |
| Death | 5,786  (5,698 to 5,874) | 6,244  (6,144 to 6,347) | 5,988  (5,898 to 6,081) |

**
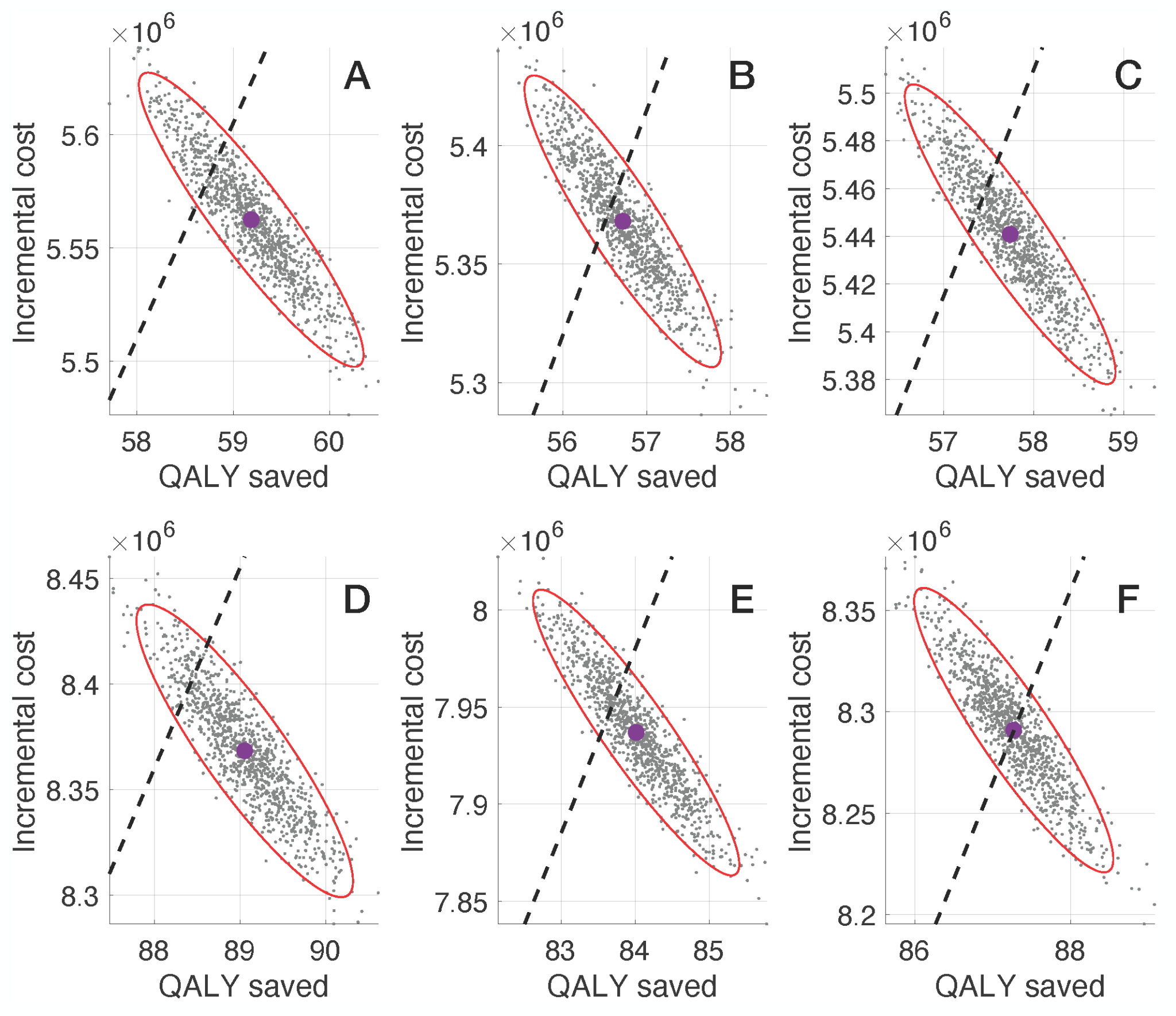
**

**Figure A6.** Cost-effectiveness planes for vaccination programs during the first RSV season with sigmoidal vaccine efficacy profiles under S1 (A,B,C) and S2 (D,E,F). Scenarios correspond to: (A) Arexvy alone with PPD of $127; (B) Abrysvo alone with PPD of $118; a combination of Arexvy and Abrysvo with PPD of $122; (D) Arexvy alone with PPD of $126; (B) Abrysvo alone with PPD of $115; and a combination of Arexvy and Abrysvo with PPD of $122. Black dashed-line corresponds to the WTP threshold of $95,000. Red curve presents the associated 95% credible ellipse of the data points distribution.

**
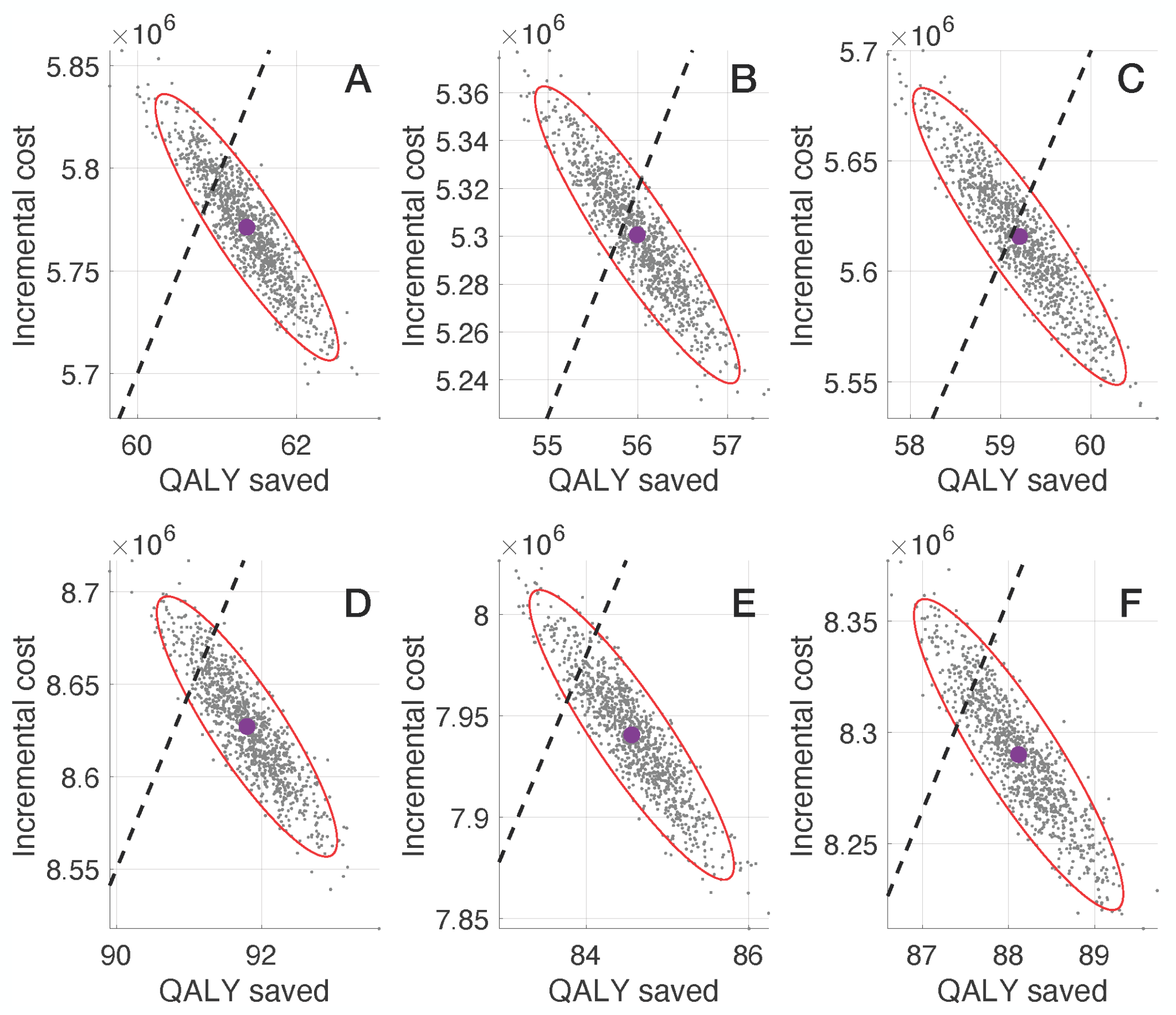
Figure A7.** Cost-effectiveness planes for vaccination programs during the first RSV season with linear vaccine efficacy profiles under S1 (A,B,C) and S2 (D,E,F). Scenarios correspond to: (A) Arexvy alone with PPD of $132; (B) Abrysvo alone with PPD of $117; a combination of Arexvy and Abrysvo with PPD of $126; (D) Arexvy alone with PPD of $130; (B) Abrysvo alone with PPD of $116; and a combination of Arexvy and Abrysvo with PPD of $123. Black dashed-line corresponds to the WTP threshold of $95,000. Red curve presents the associated 95% credible ellipse of the data points distribution.


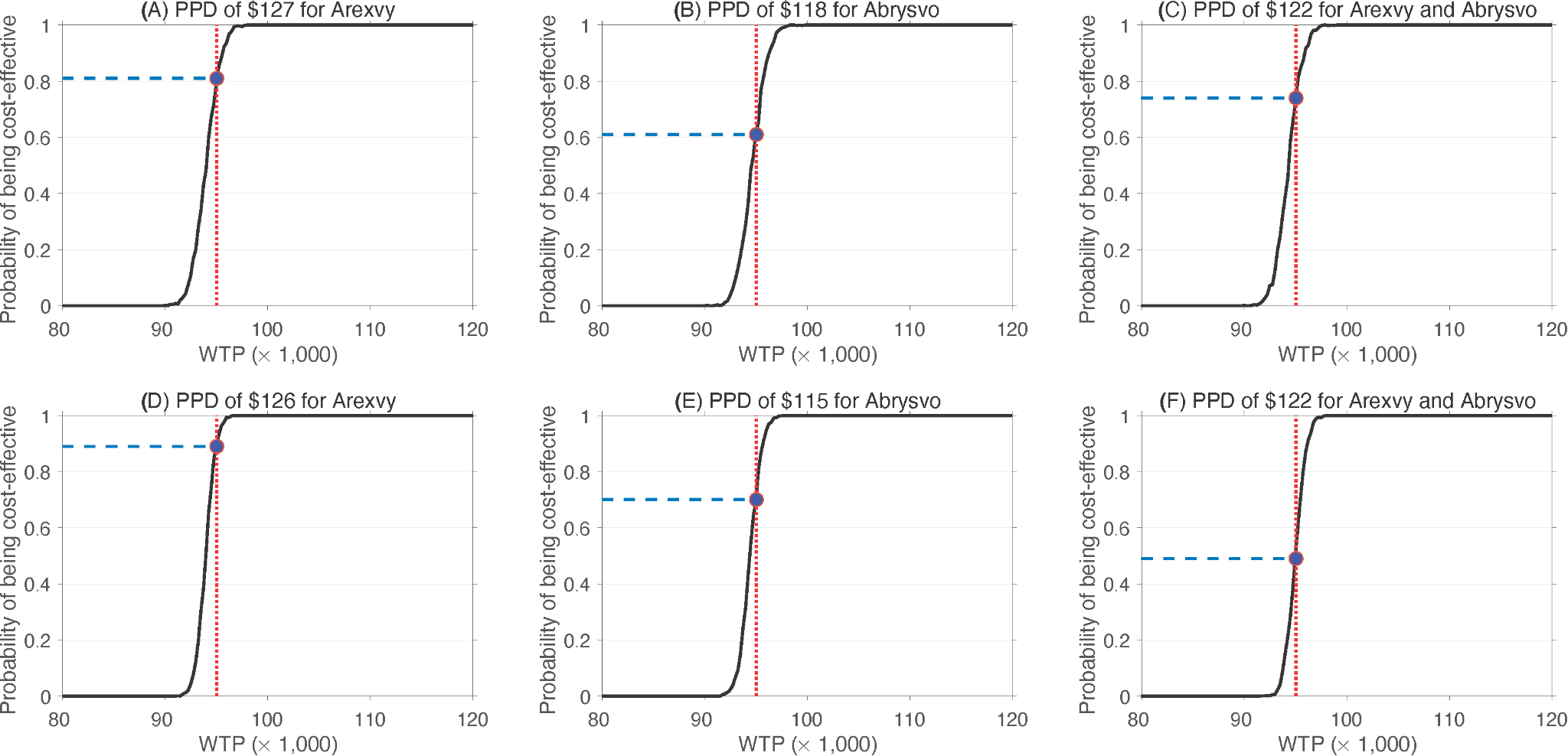
**Figure A8.** Acceptability curves for vaccination programs during the first RSV season with sigmoidal vaccine efficacy profiles under S1 (A,B,C) and S2 (D,E,F). Scenarios correspond to: (A) Arexvy alone with PPD of $127; (B) Abrysvo alone with PPD of $118; a combination of Arexvy and Abrysvo with PPD of $122; (D) Arexvy alone with PPD of $126; (B) Abrysvo alone with PPD of $115; and a combination of Arexvy and Abrysvo with PPD of $122. Red dotted-line corresponds to the WTP threshold of $95,000. Blue dashed-line presents the probability of being cost-effective for the PPD at the WTP of 95,000.


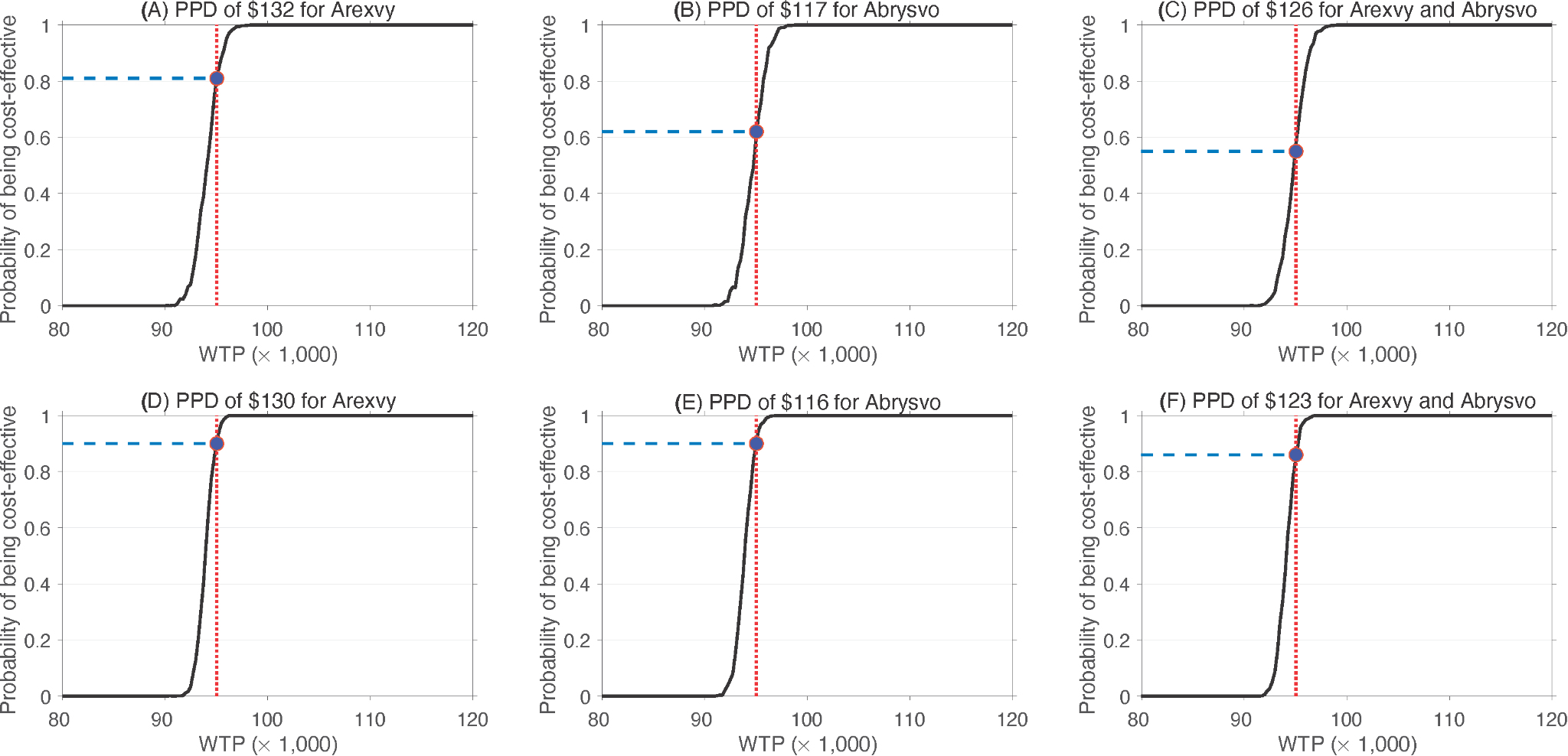


**Figure A9.** Acceptability curves for vaccination programs during the first RSV season with linear vaccine efficacy profiles under S1 (A,B,C) and S2 (D,E,F). Scenarios correspond to: (A) Arexvy alone with PPD of $132; (B) Abrysvo alone with PPD of $117; a combination of Arexvy and Abrysvo with PPD of $126; (D) Arexvy alone with PPD of $130; (B) Abrysvo alone with PPD of $116; and a combination of Arexvy and Abrysvo with PPD of $123. Red dotted-line corresponds to the WTP threshold of $95,000. Blue dashed-line presents the probability of being cost-effective for the PPD at the WTP of 95,000.

**Cost-effectiveness analysis with the WTP of $95,000 per QALY gained over two RSV seasons**

Using sigmoidal vaccine efficacy profile, S1 with 66% vaccination coverage resulted in mean reductions of 42.8%, 32.2%, and 37.5% in outpatient care using Arexvy only, Abrysvo only, and combination of Arexvy and Abrysvo, respectively (**Figure A8**). The corresponding reductions in hospitalizations were 47.4%, 47.3%, and 47.3%. Similar reductions of 44.3%, 44.1%, and 44.6% in RSV-related deaths were achieved. Increasing vaccination coverage to 100%, S2 resulted in mean reductions of 64.8%, 48.8%, and 56.9% in outpatient care; 71.9%, 71.6%, and 71.6% in hospitalizations; and 67.1%, 67.0%, and 66.7% in deaths using Arexvy only, Abrysvo only, and a combination of Arexvy and Abrysvo, respectively (**Figure A8**).


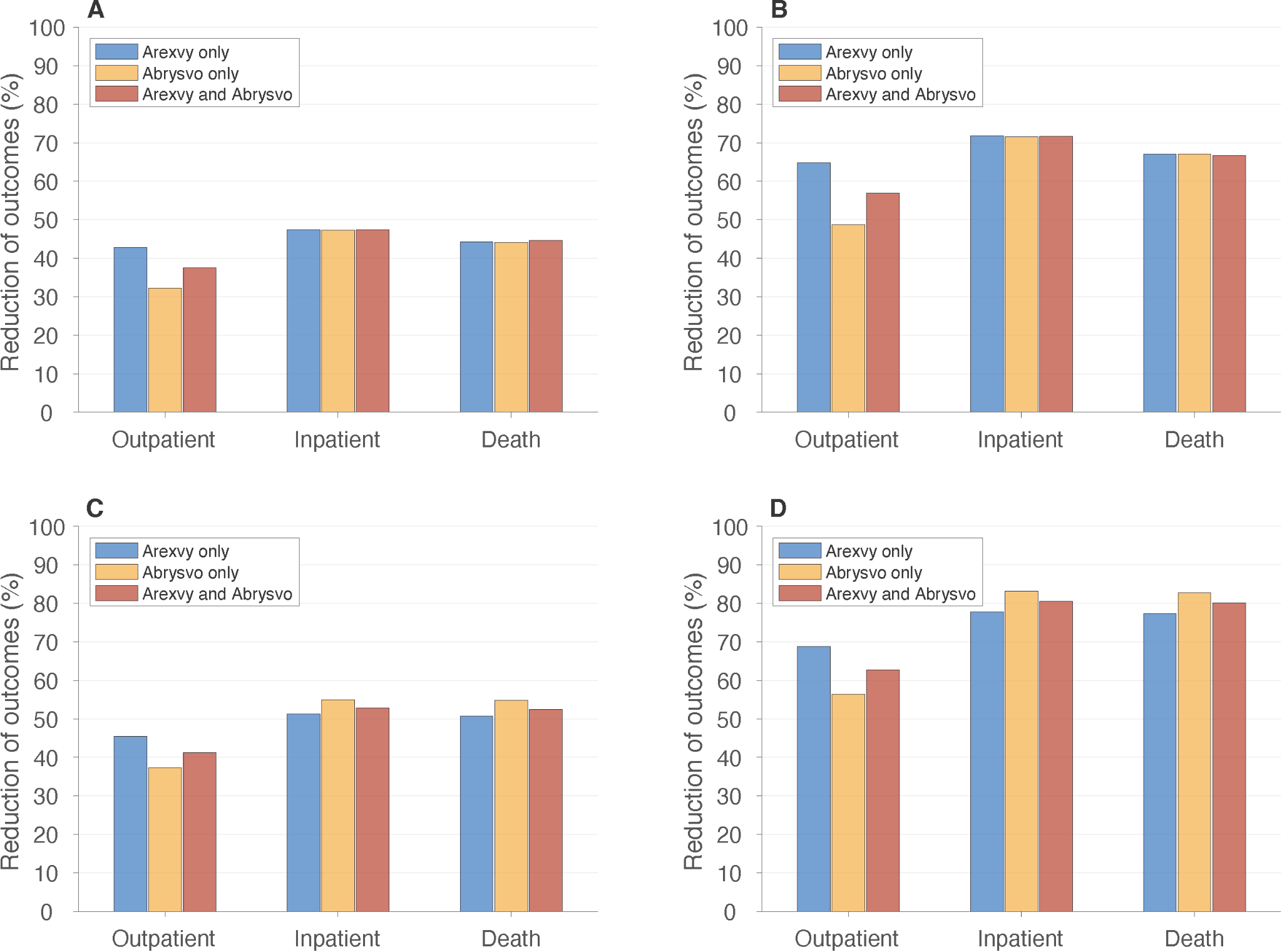
**Figure A10.** Overall reduction of RSV-related outpatient care (office and ED visits), inpatient care (hospitalization), and death among adults 60 years of age or older over two RSV seasons, compared to the scenario without vaccination, with sigmoidal (A,B) and linear (C,D) vaccine efficacy profiles. Vaccination coverage was set to 66% (A,C) and 100% (B,D).

With the linear efficacy profiles, the reduction of outcomes improved. We estimated that S1 with 66% vaccination coverage would reduce outpatient care by 45.4% using Arexvy only, by 37.3% using Abrysvo only, and by 41.3% for a combination of Arexvy and Abrysvo (**Figure A8**). The corresponding reductions in hospitalizations were 51.2%, 54.9%, and 52.8%. Similar reductions of 50.7%, 54.8%, and 52.5% in RSV-related deaths were achieved. Increasing vaccination coverage to 100%, S2 resulted in mean reductions of 68.8%, 56.5%, and 62.7% in outpatient care; 77.7%, 83.1%, and 80.5% in hospitalizations; and 77.4%, 82.7%, and 80.1% in deaths using Arexvy only, Abrysvo only, and a combination of Arexvy and Abrysvo, respectively (**Figure A8**).

Similar age-specific reductions of outcomes were achieved for S1 and S2 scenarios using sigmoidal (**Figure A9**) and linear (**Figure A10**) vaccine efficacy profiles.


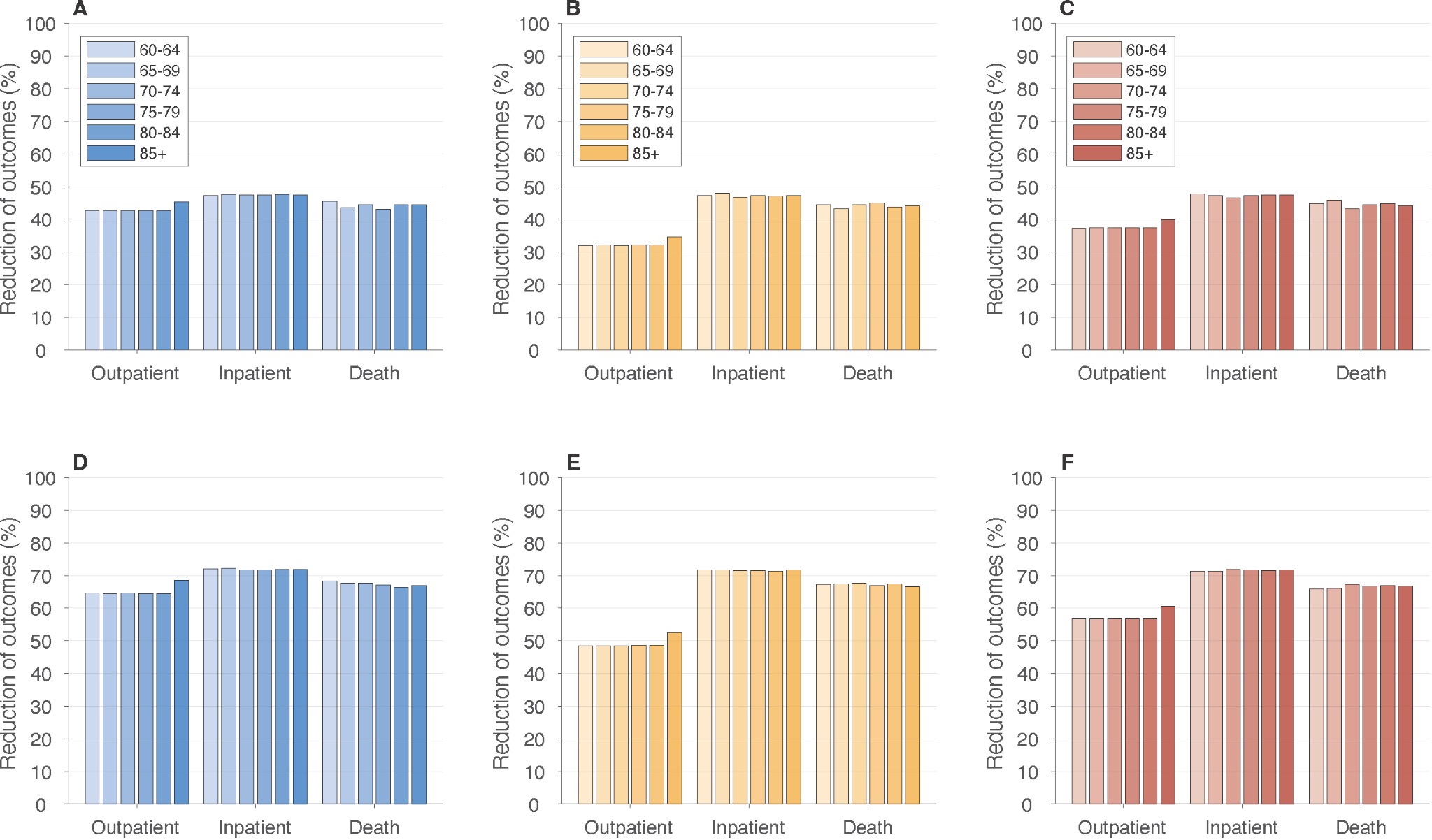
**Figure A11.** Age-specific reductions of outpatient care, hospitalization and deaths achieved in S1 with 66% vaccination coverage (A,B,C) and S2 with 100% vaccination coverage (D,E,F) over two RSV seasons. Scenarios correspond to the use of Arexvy vaccine only (A,D); Abrysvo vaccine only (B,E); and a combination of Arexvy and Abrysvo vaccines (C,F), with sigmoidal vaccine efficacy profiles.

**
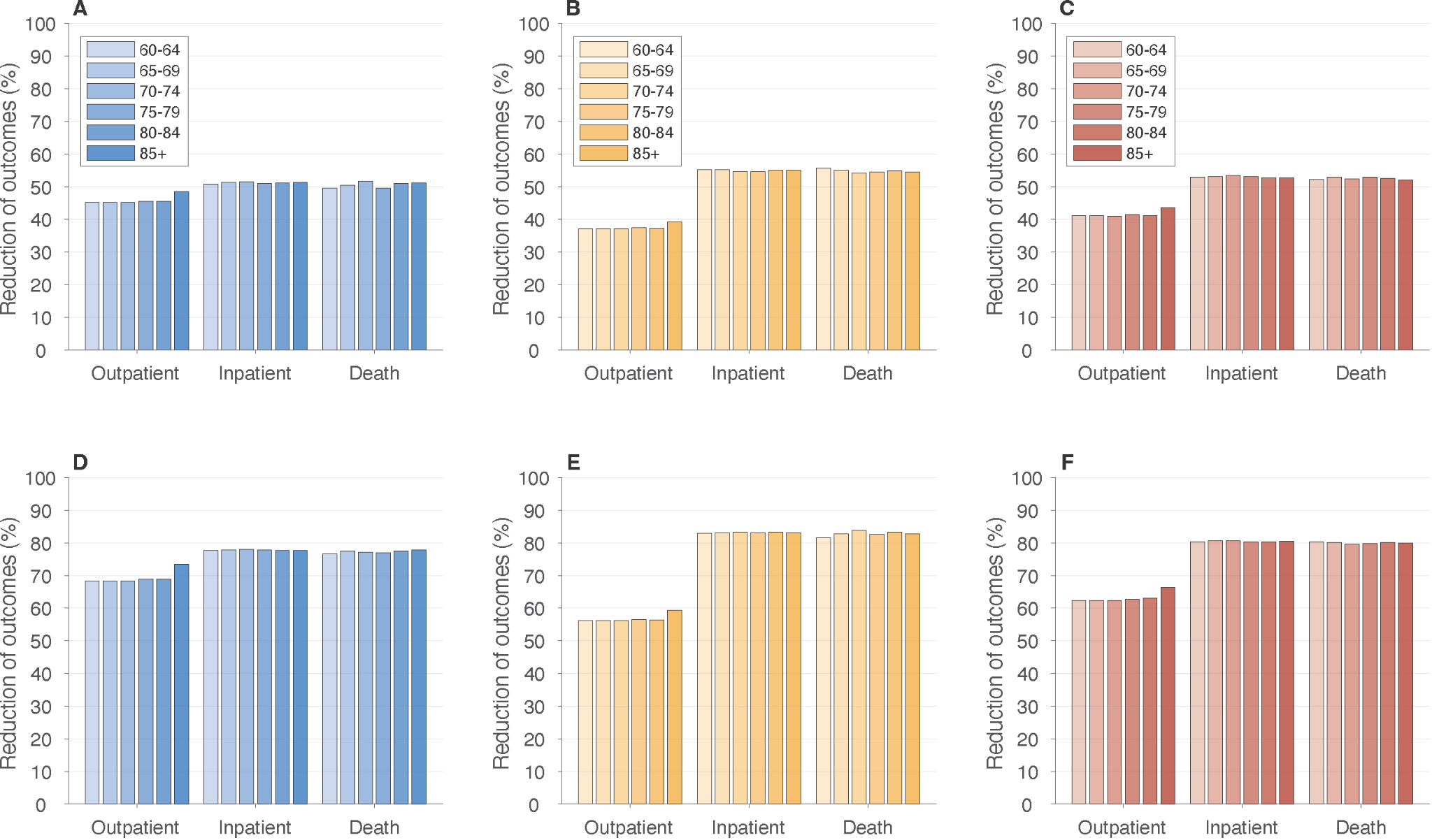
Figure A12.** Age-specific reductions of outpatient care, hospitalization and deaths achieved in S1 with 66% vaccination coverage (A,B,C) and S2 with 100% vaccination coverage (D,E,F) over two RSV seasons. Scenarios correspond to the use of Arexvy vaccine only (A,D); Abrysvo vaccine only (B,E); and a combination of Arexvy and Abrysvo vaccines (C,F), with linear vaccine efficacy profiles.

**Table A7.** Estimated net savings of RSV-related outcomes achieved over two RSV seasons using Arexvy in different age groups per 100,000 adults 60 years of age or older.

| **Age group** | **Outpatient** | **Inpatient** | **Productivity**  **(market and non-market)** |
| --- | --- | --- | --- |
| *S1 with sigmoidal vaccine efficacy* | | | |
| 60-64 | $92,729 | $105,759 | $2,273,312 |
| 65-69 | $81,140 | $119,140 | $1,167,814 |
| 70-74 | $65,895 | $101,796 | $491,666 |
| 75-79 | $42,565 | $281,757 | $575,474 |
| 80-84 | $26,049 | $176,536 | $302,601 |
| 85+ | $9,797 | $949,902 | $115,294 |
| *S2 with sigmoidal vaccine efficacy* | | | |
| 60-64 | $139,602 | $161,786 | $3,408,448 |
| 65-69 | $122,677 | $180,904 | $1,783,168 |
| 70-74 | $100,151 | $152,937 | $743,505 |
| 75-79 | $64,532 | $426,750 | $886,098 |
| 80-84 | $39,564 | $265,298 | $450,922 |
| 85+ | $14,432 | $1,440,853 | $172,166 |
| *S1 with linear vaccine efficacy* | | | |
| 60-64 | $98,611 | $111,438 | $2,408,132 |
| 65-69 | $86,547 | $129,371 | $1,308,739 |
| 70-74 | $70,377 | $114,406 | $540,884 |
| 75-79 | $45,774 | $307,911 | $643,495 |
| 80-84 | $27,988 | $190,974 | $337,986 |
| 85+ | $10,485 | $1,043,033 | $129,109 |
| *S2 with linear vaccine efficacy* | | | |
| 60-64 | $148,558 | $173,930 | $3,718,050 |
| 65-69 | $130,056 | $196,033 | $1,992,976 |
| 70-74 | $105,860 | $168,412 | $838,685 |
| 75-79 | $68,713 | $468,487 | $990,041 |
| 80-84 | $42,162 | $290,525 | $515,338 |
| 85+ | $15,425 | $1,585,576 | $198,774 |

**Table A8.** Estimated net savings of RSV-related outcomes achieved over two RSV seasons using Abrysvo in different age groups per 100,000 adults 60 years of age or older.

| **Age group** | **Outpatient** | **Inpatient** | **Productivity**  **(market and non-market)** |
| --- | --- | --- | --- |
| *S1 with sigmoidal vaccine efficacy* | | | |
| 60-64 | $68,947 | $104,236 | $2,042,091 |
| 65-69 | $60,505 | $121,176 | $1,044,553 |
| 70-74 | $48,983 | $99,175 | $424,155 |
| 75-79 | $31,172 | $280,878 | $548,100 |
| 80-84 | $19,127 | $172,659 | $278,928 |
| 85+ | $3,994 | $947,957 | $108,383 |
| *S2 with sigmoidal vaccine efficacy* | | | |
| 60-64 | $104,827 | $160,645 | $3,081,446 |
| 65-69 | $91,850 | $179,456 | $1,635,730 |
| 70-74 | $74,909 | $150,839 | $643,403 |
| 75-79 | $47,365 | $425,197 | $828,091 |
| 80-84 | $28,894 | $260,426 | $429,041 |
| 85+ | $6,262 | $1,433,233 | $166,339 |
| *S1 with linear vaccine efficacy* | | | |
| 60-64 | $80,223 | $123,155 | $2,453,659 |
| 65-69 | $70,217 | $139,055 | $1,294,066 |
| 70-74 | $56,930 | $121,100 | $509,940 |
| 75-79 | $36,363 | $327,988 | $652,781 |
| 80-84 | $22,153 | $206,633 | $342,343 |
| 85+ | $4,394 | $1,114,801 | $132,347 |
| *S2 with linear vaccine efficacy* | | | |
| 60-64 | $122,298 | $185,662 | $3,663,608 |
| 65-69 | $107,171 | $211,017 | $1,985,932 |
| 70-74 | $87,148 | $182,928 | $764,574 |
| 75-79 | $55,352 | $500,026 | $1,011,011 |
| 80-84 | $33,706 | $319,286 | $522,353 |
| 85+ | $6,811 | $1,700,262 | $203,230 |

*Number needed to vaccinate (NNV)*

The mean number of adults in the study population who needed to be vaccinated to avert one outpatient over two RSV seasons with sigmoidal vaccine efficacy profiles ranged from 33 to 44 (**Table A9**). Mean estimated NNV to avert one hospitalization ranged from 325 to 326, and from 3,977 to 4,043 to prevent one death. With the linear vaccine efficacy profiles, NNV to avert one outpatient visit ranged from 31 to 38; to avert one hospitalization ranged from 281 to 301; and to avert one death ranged from 3,277 to 3,536 (**Table A9**).

**Table A9.** Number of vaccine doses needed to avert one outcome over two RSV seasons with temporal vaccine efficacies using sigmoidal fit and linear average estimates (**Figures A2**).

| **Outcome** | **NNV to avert one outcome: mean (95% CI)** | | |
| --- | --- | --- | --- |
|  | **Arexvy only** | **Abrysvo only** | **Arexvy and Abrysvo** |
| *Sigmoidal vaccine efficacy* | | | |
| Outpatient | 33  (33 to 33) | 44  (43 to 44) | 38  (37 to 38) |
| Hospitalization | 325  (323 to 327) | 326  (324 to 328) | 326  (324 to 328) |
| Death | 3,984  (3,926 to 4,043) | 4,043  (3,984 to 4,104) | 3,977  (3,918 to 4,036) |
| *Linear vaccine efficacy* | | | |
| Outpatient | 31  (30 to 31) | 38  (38 to 38) | 34  (34 to 35) |
| Hospitalization | 301  (300 to 303) | 281  (280 to 283) | 291  (290 to 292) |
| Death | 3,536  (3,488 to 3,586) | 3,277  (3,231 to 3,321) | 3,384  (3,337 to 3,431) |


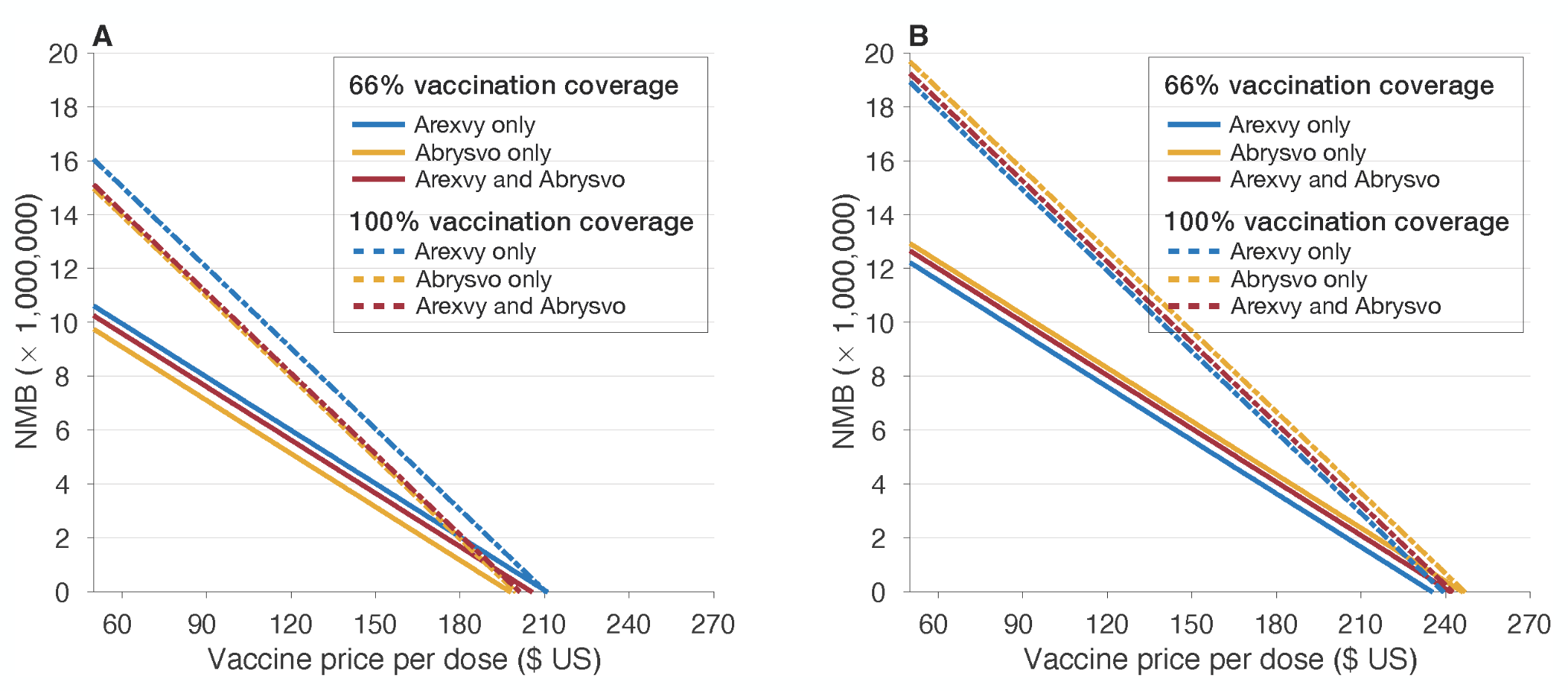
**Figure A13.** Estimated net monetary benefit (NMB) over two RSV seasons as a function of price per dose for Arexvy and Abrysvo with different coverage of vaccination, and sigmoidal (A) and linear (B) vaccine efficacy profiles. For scenarios using both Arexvy and Abrysvo, each vaccine was assumed to have 50% of the target coverage with the same price per dose.

**Table A10.** Model estimates of cost-effectiveness analyses for vaccination programs with Arexvy only, Abrysvo only, and combination of Arexvy and Abrysvo over two RSV seasons in a population of 100,000 adults aged 60 years or older at the WTP of $95,000. All strategies were compared to the baseline with no intervention.

| **Scenario** | **Maximum**  **PPD, $** | **Incremental costs, $**  **(95% CI)** | **QALY saved**  **(95% CI)** | **ICER**  **(95% CI)** | **Probability of being cost-effective** | **Budget impact per 100,000, $** | **National budget impact, $ billion** |
| --- | --- | --- | --- | --- | --- | --- | --- |
| *S1 with sigmoidal vaccine efficacy* | | | | | | | |
| Arexvy only | 210 | 8,530,775  (8,457,609 to 8,603,687) | 90.34  (88.95 to 91.68) | 94,432  (92,237 to 96,687) | 70% | 13,457,126 | 10.62 |
| Abrysvo only | 197 | 8,246,983  (8,178,851 to 8,315,116) | 87.25  (85.96 to 88.58) | 94,522  (92,367 to 96,689) | 67% | 12,693,137 | 10.02 |
| Arexvy and Abrysvo | 205 | 8,496,981  (8,429,066 to 8,566,003) | 89.56  (88.18 to 90.88) | 94,879  (92,815 to 97,143) | 53% | 13,180,646 | 10.40 |
| *S2 with sigmoidal vaccine efficacy* | | | | | | | |
| Arexvy only | 210 | 12,946,207  (12,853,092 to 13,029,062) | 136.62  (135.11 to 138.38) | 94,760  (92,922 to 96,430) | 59% | 20,390,119 | 16.09 |
| Abrysvo only | 199 | 12,652,045  (12,575,988 to 12,729,776) | 133.82  (132.30 to 135.33) | 94,546  (92,992 to 96,163) | 71% | 19,436,450 | 15.34 |
| Arexvy and Abrysvo | 201 | 12,587,845  (12,510,078 to 12,670,292) | 132.54  (131.00 to 133.99) | 94,977  (93,385 to 96,670) | 52% | 19,566,018 | 15.44 |
| *S1 with linear vaccine efficacy* | | | | | | | |
| Arexvy only | 235 | 9,554,741  (9,471,770 to 9,625,600) | 100.63  (99.27 to 102.16) | 94,949  (92,830 to 96,884) | 51% | 14,922,770 | 11.78 |
| Abrysvo only | 245 | 10,131,852  (10,060,772 to 10,209,639) | 107.25  (105.82 to 108.59) | 94,465  (92,646 to 96,456) | 72% | 15,517,549 | 12.25 |
| Arexvy and Abrysvo | 241 | 9,894,996  (9,821,395 to 9,965,363) | 104.64  (103.18 to 106.03) | 94,565  (92,689 to 96,569) | 67% | 15,288,999 | 12.07 |
| *S2 with linear vaccine efficacy* | | | | | | | |
| Arexvy only | 239 | 14,752,398  (14,668,805 to 14,835,653) | 155.43  (153.76 to 157.05) | 94,912  (93,434 to 96,467) | 54% | 23,006,286 | 18.16 |
| Abrysvo only | 246 | 15,437,625  (15,347,222 to 15,533,217) | 163.26  (161.41 to 165.05) | 94,559  (92,972 to 96,235) | 72% | 23,588,727 | 18.61 |
| Arexvy and Abrysvo | 242 | 15,033,949  (14,946,899 to 15,119,611) | 158.58  (156.89 to 160.30) | 94,804  (93,230 to 96,348) | 59% | 23,250,529 | 18.35 |


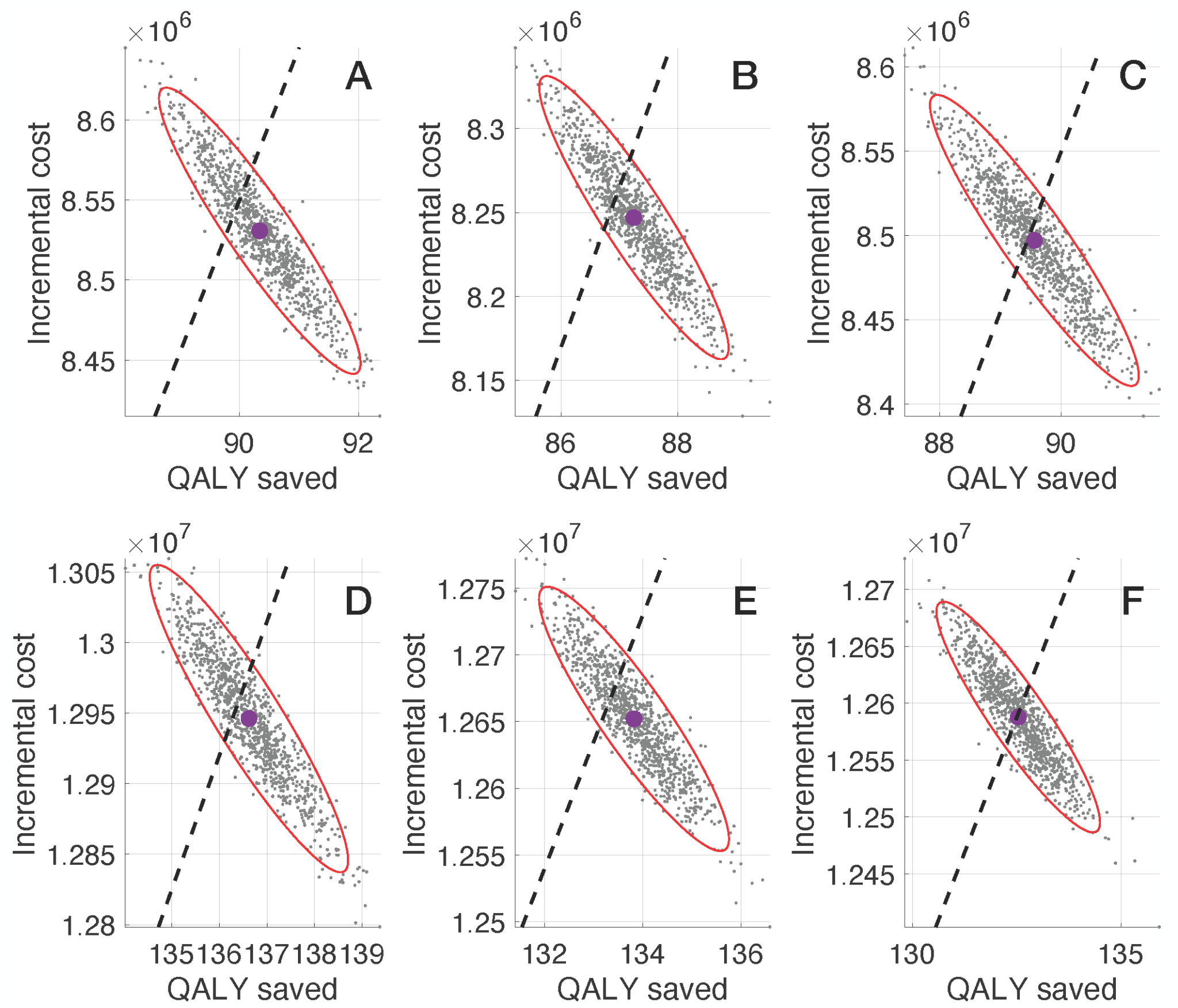
**Figure A14.** Cost-effectiveness planes for vaccination programs over two RSV seasons with sigmoidal vaccine efficacy profiles under S1 (A,B,C) and S2 (D,E,F). Scenarios correspond to: (A) Arexvy alone with PPD of $210; (B) Abrysvo alone with PPD of $197; a combination of Arexvy and Abrysvo with PPD of $205; (D) Arexvy alone with PPD of $210; (B) Abrysvo alone with PPD of $199; and a combination of Arexvy and Abrysvo with PPD of $201. Black dashed-line corresponds to the WTP threshold of $95,000. Red curve presents the associated 95% credible ellipse of the data points distribution.


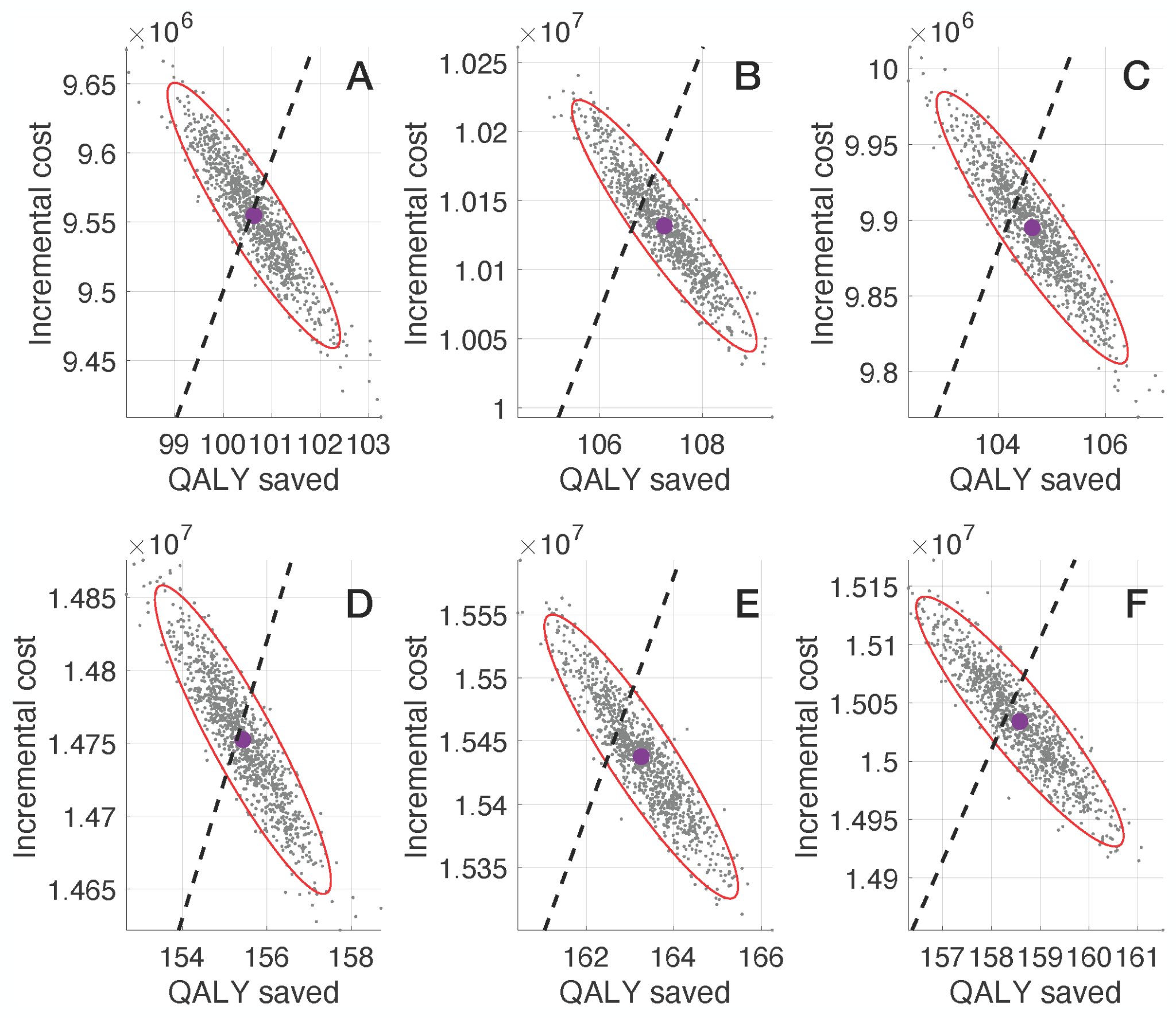
**Figure A15.** Cost-effectiveness planes for vaccination programs over two RSV seasons with linear vaccine efficacy profiles under S1 (A,B,C) and S2 (D,E,F). Scenarios correspond to: (A) Arexvy alone with PPD of $235; (B) Abrysvo alone with PPD of $245; a combination of Arexvy and Abrysvo with PPD of $241; (D) Arexvy alone with PPD of $239; (B) Abrysvo alone with PPD of $246; and a combination of Arexvy and Abrysvo with PPD of $242. Black dashed-line corresponds to the WTP threshold of $95,000. Red curve presents the associated 95% credible ellipse of the data points distribution.


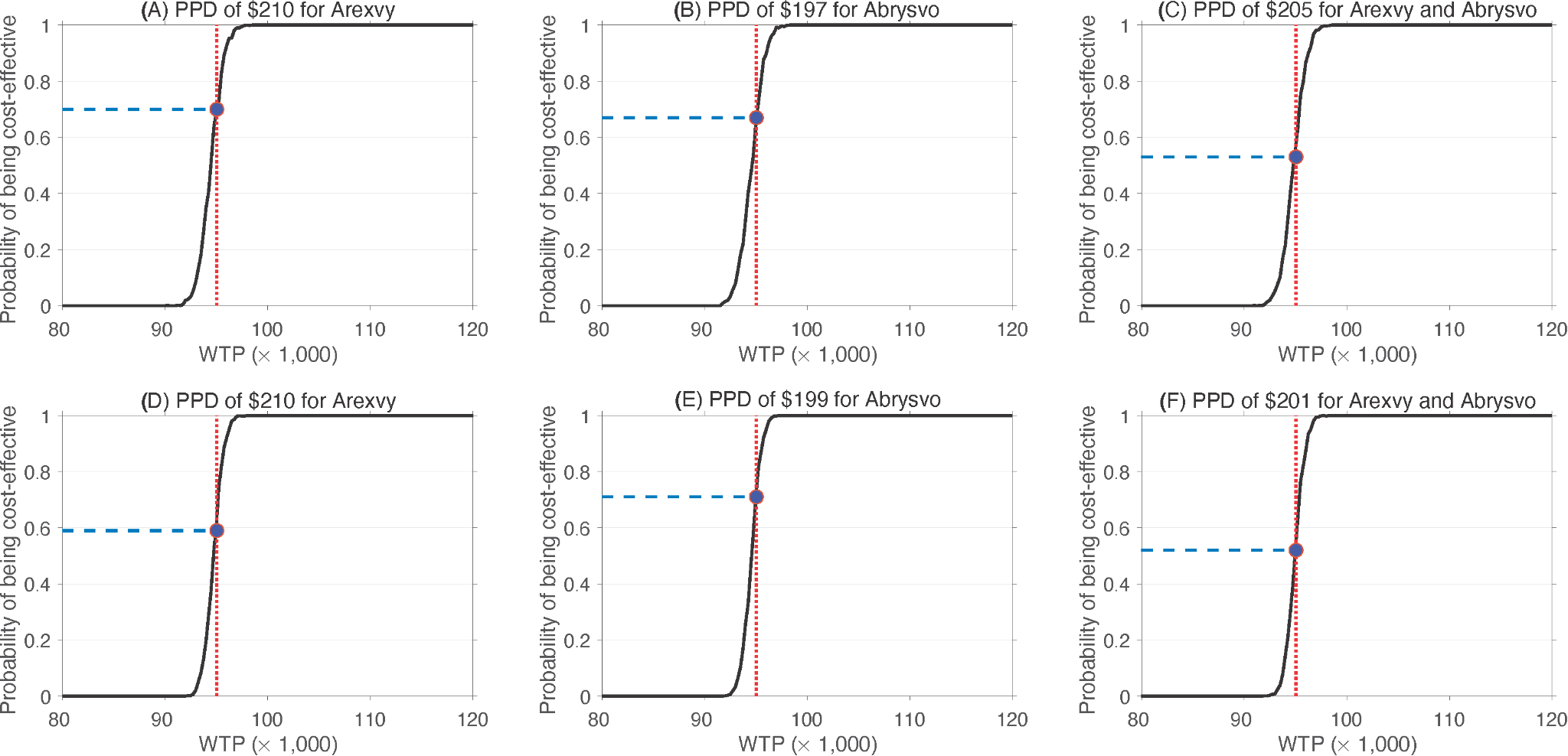
**Figure A16.** Acceptability curves for vaccination programs over two RSV seasons with sigmoidal vaccine efficacy profiles under S1 (A,B,C) and S2 (D,E,F). Scenarios correspond to: (A) Arexvy alone with PPD of $210; (B) Abrysvo alone with PPD of $197; a combination of Arexvy and Abrysvo with PPD of $205; (D) Arexvy alone with PPD of $210; (B) Abrysvo alone with PPD of $199; and a combination of Arexvy and Abrysvo with PPD of $201. Red dotted-line corresponds to the WTP threshold of $95,000. Blue dashed-line presents the probability of being cost-effective for the PPD at the WTP of 95,000.


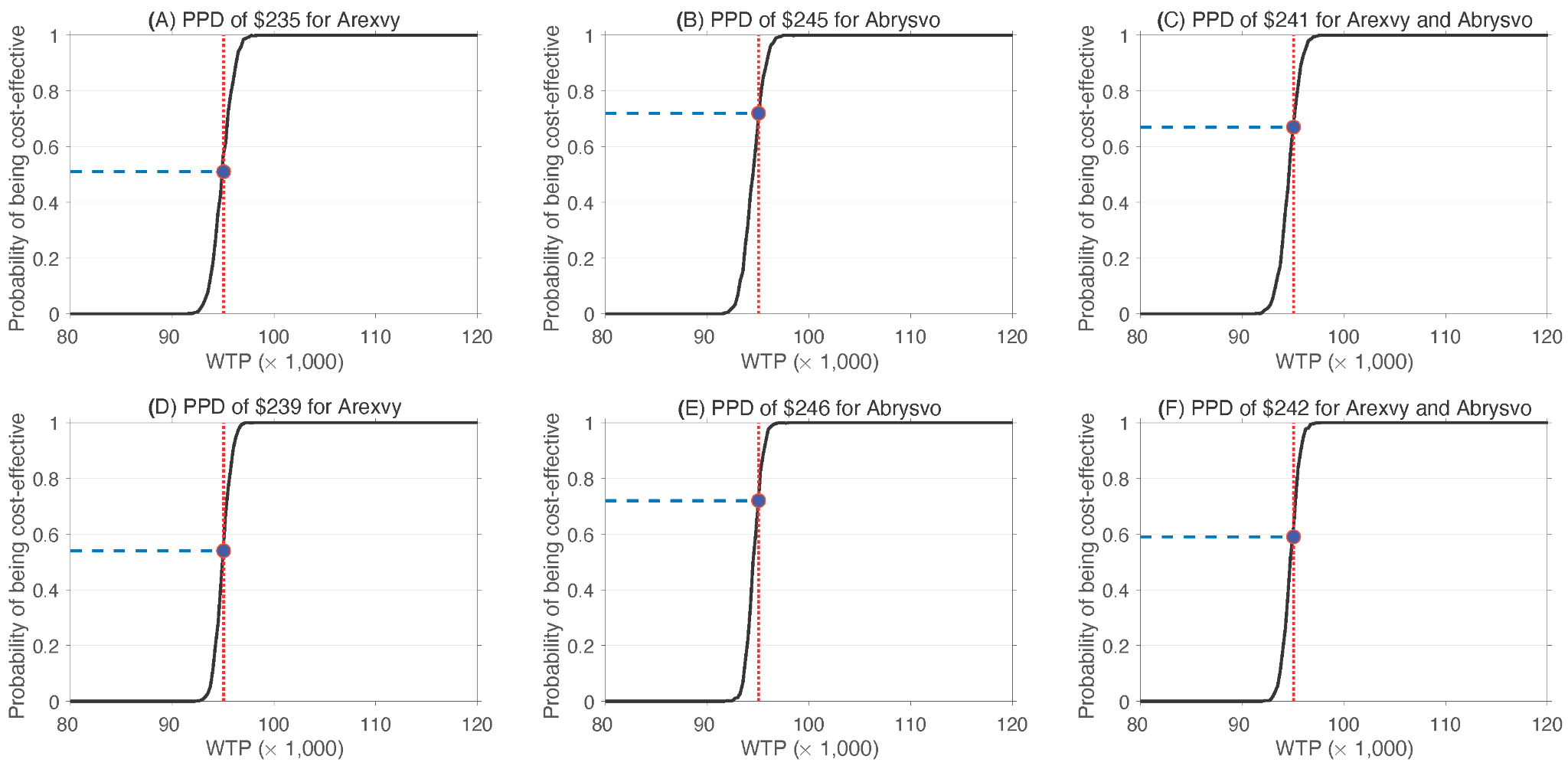
**Figure A17.** Acceptability curves for vaccination programs over two RSV seasons with linear vaccine efficacy profiles under S1 (A,B,C) and S2 (D,E,F). Scenarios correspond to: (A) Arexvy alone with PPD of $235; (B) Abrysvo alone with PPD of $245; a combination of Arexvy and Abrysvo with PPD of $241; (D) Arexvy alone with PPD of $239; (B) Abrysvo alone with PPD of $246; and a combination of Arexvy and Abrysvo with PPD of $242. Red dotted-line corresponds to the WTP threshold of $95,000. Blue dashed-line presents the probability of being cost-effective for the PPD at the WTP of 95,000.

**Cost-effectiveness analysis with the WTP of $80,000 per QALY gained during the first RSV season**

**
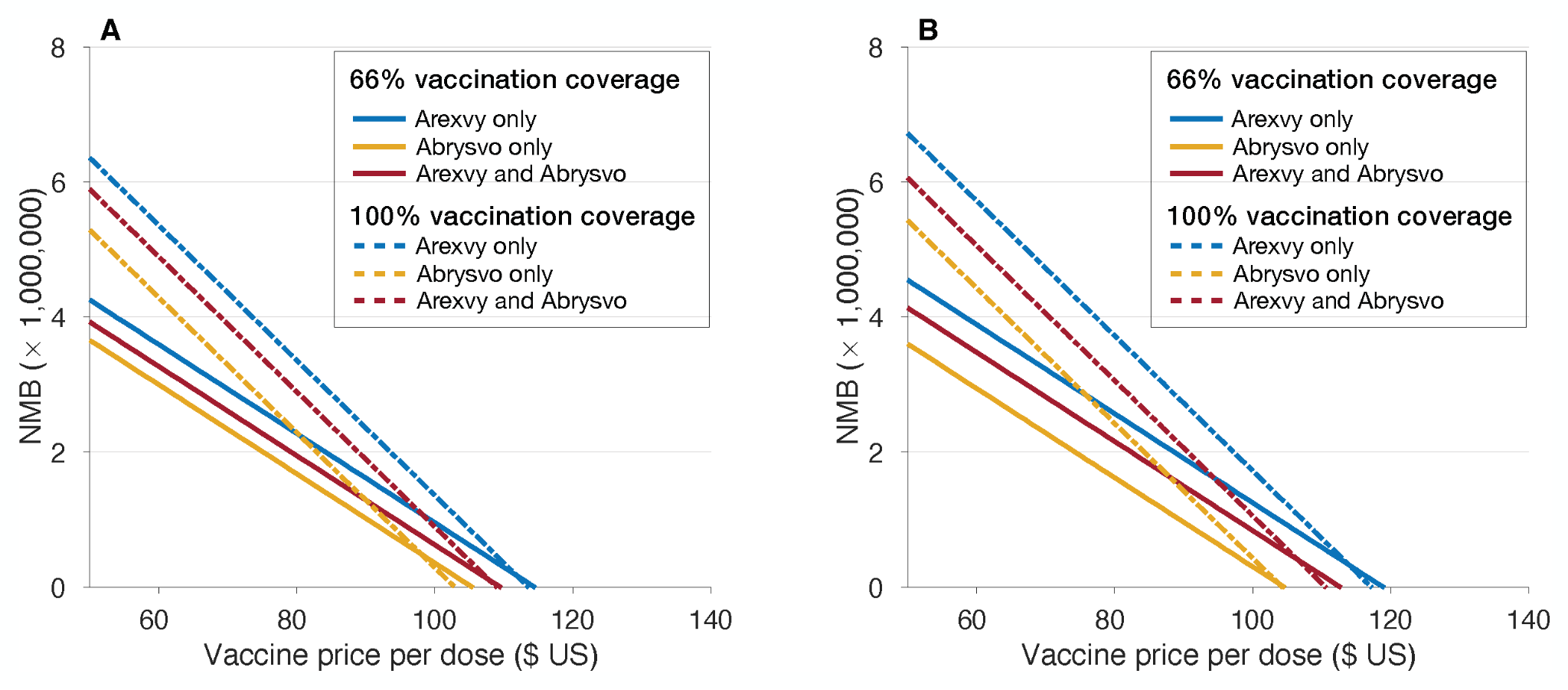
Figure A18.** Estimated net monetary benefit (NMB) during the first RSV season as a function of price per dose for Arexvy and Abrysvo with different coverage of vaccination, and sigmoidal (A) and linear (B) vaccine efficacy profiles. For scenarios using both Arexvy and Abrysvo, each vaccine was assumed to have 50% of the target coverage with the same price per dose.

**Table A11.** Model estimates of cost-effectiveness analyses for vaccination programs with Arexvy only, Abrysvo only, and combination of Arexvy and Abrysvo during the first RSV season in a population of 100,000 adults aged 60 years or older at the WTP of $80,000. All strategies were compared to the baseline with no intervention.

| **Scenario** | **Maximum**  **PPD, $** | **Incremental costs, $**  **(95% CI)** | **QALY saved**  **(95% CI)** | **ICER**  **(95% CI)** | **Probability of being cost-effective** | **Budget impact per 100,000, $** | **National budget impact, $ billion** |
| --- | --- | --- | --- | --- | --- | --- | --- |
| *S1 with sigmoidal vaccine efficacy* | | | | | | | |
| Arexvy only | 114 | 4,704,363  (4,652,302 to 4,756,552) | 59.19  (58.25 to 60.14) | 79,484  (77,376 to 81,633) | 68% | 7,841,485 | 6.19 |
| Abrysvo only | 105 | 4,510,121  (4,463,784 to 4,558,761) | 56.71  (55.82 57.67) | 79,523  (77,398 to 81,558) | 67% | 7,351,939 | 5.80 |
| Arexvy and Abrysvo | 109 | 4,582,866  (4,530,861 to 4,633,542) | 57.74  (56.78 to 58.72) | 79,374  (77,221 to 81,509) | 72% | 7,571,527 | 5.97 |
| *S2 with sigmoidal vaccine efficacy* | | | | | | | |
| Arexvy only | 113 | 7,068,448  (7,014,923 to 7,124,116) | 89.06  (88.09 to 90.09) | 79,370  (77,915 to 80,842) | 79% | 11,792,291 | 9.31 |
| Abrysvo only | 102 | 6,636,899  (6,576,114 to 6,695,439) | 84.01  (82.87 to 85.10) | 78,998  (77,332 to 80,730) | 87% | 10,849,858 | 8.56 |
| Arexvy and Abrysvo | 108 | 6,890,977  (6,832,919 to 6,948,981) | 87.27  (86.23 to 88.31) | 78,962  (77,438 to 80,554) | 91% | 11,379,360 | 8.98 |
| *S1 with linear vaccine efficacy* | | | | | | | |
| Arexvy only | 118 | 4,847,240  (4,794,678 to 4,897,915) | 61.37  (60.45 to 62.31) | 78,979  (76,919 to 81,018) | 85% | 8,090,880 | 6.38 |
| Abrysvo only | 104 | 4,442,596  (4,394,638 to 4,491,812) | 55.99  (55.06 to 56.87) | 79,341  (77,280 to 81,559) | 72% | 7,279,178 | 5.74 |
| Arexvy and Abrysvo | 112 | 4,691,828  (4,639,478 to 4,748,281) | 59.21  (58.21 to 60.15) | 79,243  (77,185 to 81,531) | 75% | 7,756,022 | 6.12 |
| *S2 with linear vaccine efficacy* | | | | | | | |
| Arexvy only | 117 | 7,327,058  (7,271,284 to 7,385,392) | 91.80  (90.77 to 92.78) | 79,816  (78,405 to 81,339) | 61% | 12,161,485 | 9.60 |
| Abrysvo only | 104 | 6,740,563  (6,680,804 to 6,797,330) | 84.56  (83.48 to 85.55) | 79,715  (78,112 to 81,371) | 63% | 11,032,195 | 8.71 |
| Arexvy and Abrysvo | 110 | 6,990,016  (6,934,388 to 7,045,572) | 88.12  (87.15 to 89.10) | 79,328  (77,848 to 80,830) | 80% | 11,542,803 | 9.11 |

**
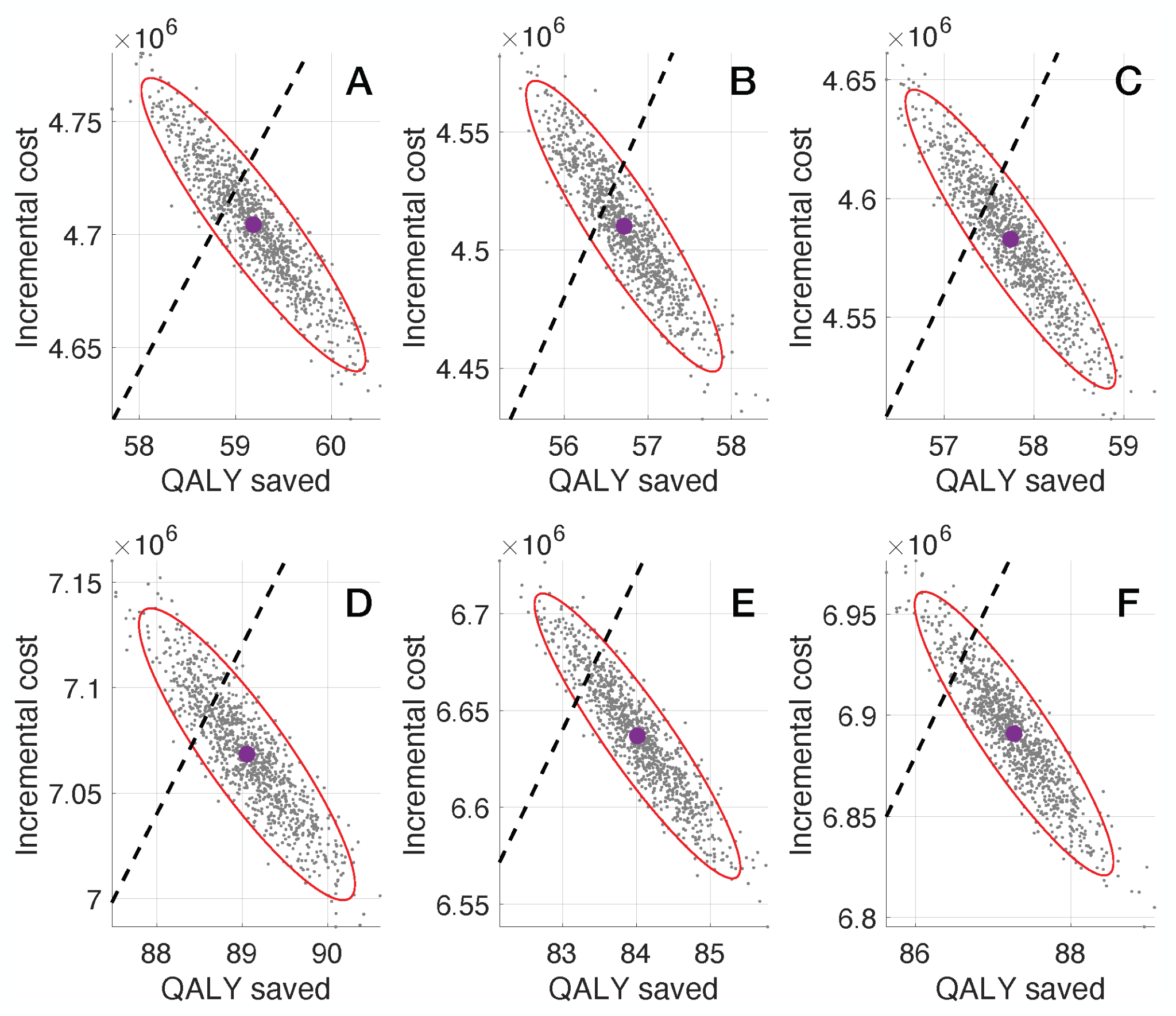
Figure A19.** Cost-effectiveness planes for vaccination programs during the first RSV season with sigmoidal vaccine efficacy profiles under S1 (A,B,C) and S2 (D,E,F). Scenarios correspond to: (A) Arexvy alone with PPD of $114; (B) Abrysvo alone with PPD of $105; a combination of Arexvy and Abrysvo with PPD of $109; (D) Arexvy alone with PPD of $113; (B) Abrysvo alone with PPD of $102; and a combination of Arexvy and Abrysvo with PPD of $108. Black dashed-line corresponds to the WTP threshold of $80,000. Red curve presents the associated 95% credible ellipse of the data points distribution.

**
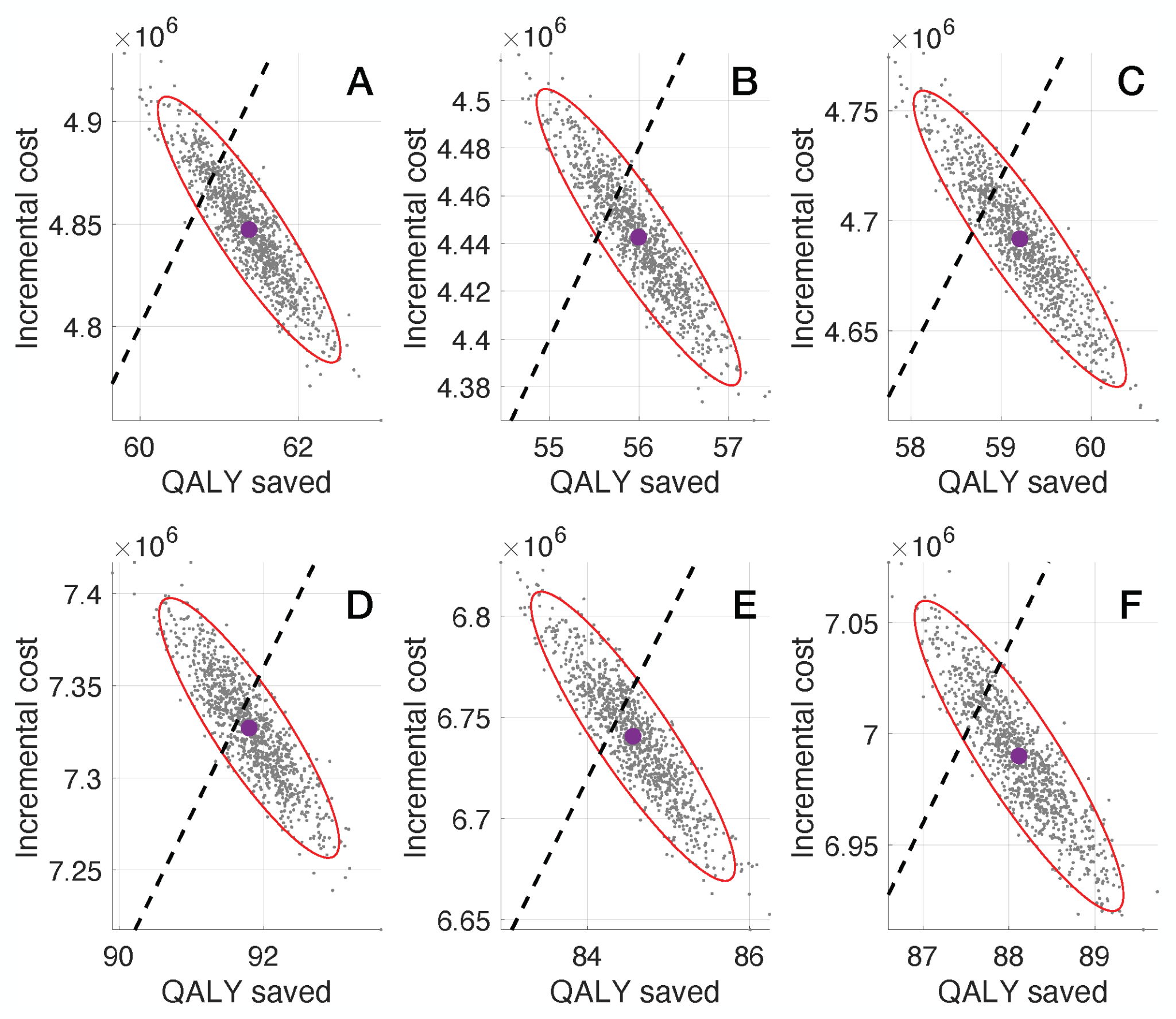
Figure A20.** Cost-effectiveness planes for vaccination programs during the first RSV season with linear vaccine efficacy profiles under S1 (A,B,C) and S2 (D,E,F). Scenarios correspond to: (A) Arexvy alone with PPD of $118; (B) Abrysvo alone with PPD of $104; a combination of Arexvy and Abrysvo with PPD of $112; (D) Arexvy alone with PPD of $117; (B) Abrysvo alone with PPD of $104; and a combination of Arexvy and Abrysvo with PPD of $110. Black dashed-line corresponds to the WTP threshold of $80,000. Red curve presents the associated 95% credible ellipse of the data points distribution.

**Cost-effectiveness analysis with the WTP of $80,000 per QALY gained over two RSV seasons**

**
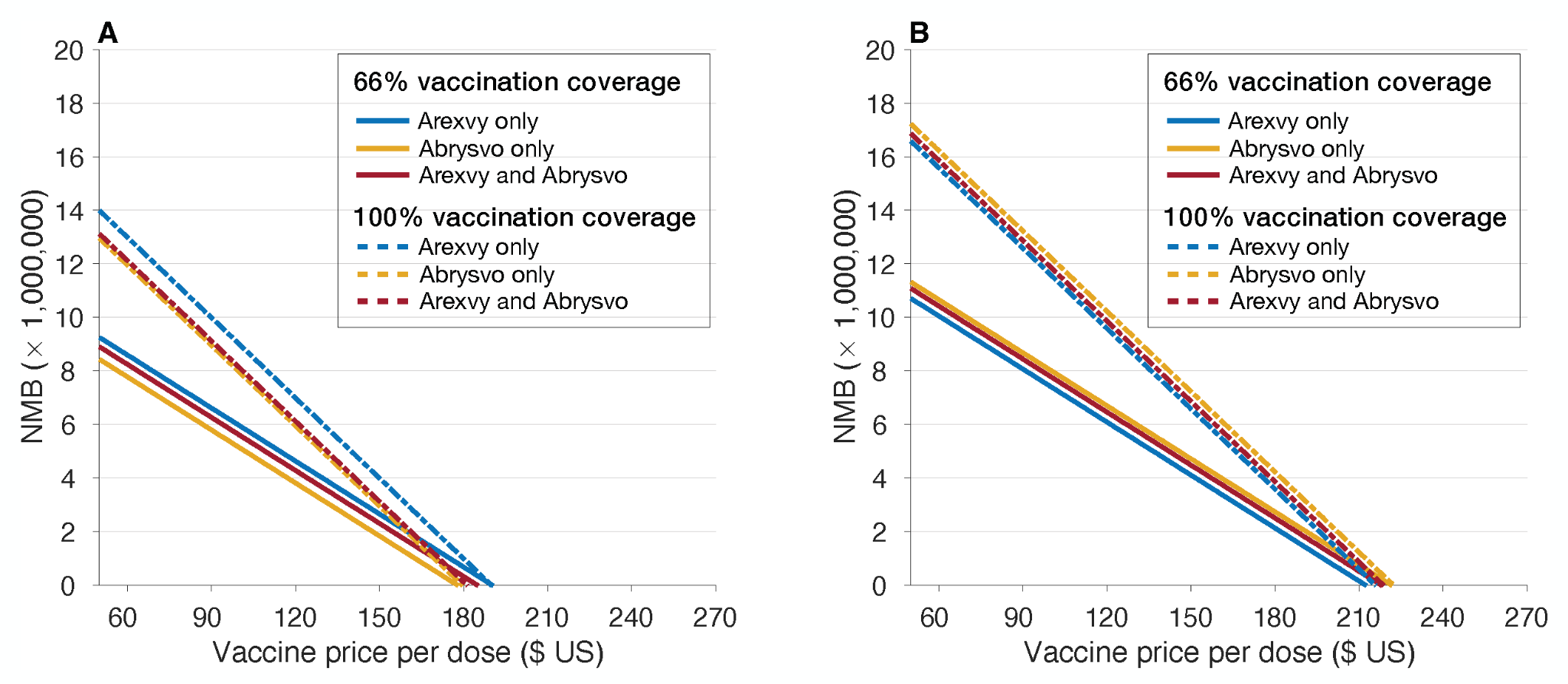
Figure A21.** Estimated net monetary benefit (NMB) over two RSV seasons as a function of price per dose for Arexvy and Abrysvo with different coverage of vaccination, and sigmoidal (A) and linear (B) vaccine efficacy profiles. For scenarios using both Arexvy and Abrysvo, each vaccine was assumed to have 50% of the target coverage with the same price per dose.

**Table A12.** Model estimates of cost-effectiveness analyses for vaccination programs with Arexvy only, Abrysvo only, and combination of Arexvy and Abrysvo over two RSV seasons in a population of 100,000 adults aged 60 years or older at the WTP of $80,000. All strategies were compared to the baseline with no intervention.

| **Scenario** | **Maximum**  **PPD, $** | **Incremental costs, $**  **(95% CI)** | **QALY saved**  **(95% CI)** | **ICER**  **(95% CI)** | **Probability of being cost-effective** | **Budget impact per 100,000, $** | **National budget impact, $ billion** |
| --- | --- | --- | --- | --- | --- | --- | --- |
| *S1 with sigmoidal vaccine efficacy* | | | | | | | |
| Arexvy only | 190 | 7,210,775  (7,137,609 to 7,283,687) | 90.34  (88.95 to 91.68) | 79,820  (77,860 to 81,850) | 58% | 12,137,126 | 9.58 |
| Abrysvo only | 177 | 6,926,983  (6,858,851 to 6,995,116) | 87.25  (85.96 to 88.58) | 79,393  (77,447 to 81,340) | 73% | 11,373,137 | 8.97 |
| Arexvy and Abrysvo | 184 | 7,110,981  (7,043,066 to 7,180,003) | 89.56  (88.18 to 90.88) | 79,403  (77,554 to 81,426) | 72% | 11,794,646 | 9.31 |
| *S2 with sigmoidal vaccine efficacy* | | | | | | | |
| Arexvy only | 189 | 10,846,207  (10,753,092 to 10,929,062) | 136.62  (135.11 to 138.38) | 79,389  (77,730 to 80,884) | 78% | 18,290,119 | 14.43 |
| Abrysvo only | 179 | 10,652,045  (10,575,988 to 10,729,776) | 133.82  (132.30 to 135.33) | 79,601  (78,210 to 81,051) | 71% | 17,436,450 | 13.76 |
| Arexvy and Abrysvo | 181 | 10,587,845  (10,510,078 to 10,670,292) | 132.54  (131.00 to 133.99) | 79,887  (78,457 to 81,412) | 57% | 17,566,018 | 13.86 |
| *S1 with linear vaccine efficacy* | | | | | | | |
| Arexvy only | 212 | 8,036,741  (7,953,770 to 8,107,600) | 100.63  (99.27 to 102.16) | 79,864  (77,955 to 81,601) | 55% | 13,404,770 | 10.58 |
| Abrysvo only | 221 | 8,547,852  (8,476,772 to 8,625,639) | 107.25  (105.82 to 108.59) | 79,697  (78,060 to 81,491) | 65% | 13,933,549 | 11.00 |
| Arexvy and Abrysvo | 217 | 8,310,996  (8,237,395 to 8,381,363) | 104.64  (103.18 to 106.03) | 79,427  (77,745 to 81,218) | 73% | 13,704,999 | 10.82 |
| *S2 with linear vaccine efficacy* | | | | | | | |
| Arexvy only | 215 | 12,352,398  (12,268,805 to 12,435,653) | 155.43  (153.76 to 157.05) | 79,471  (78,144 to 80,854) | 78% | 20,606,286 | 16.26 |
| Abrysvo only | 222 | 13,037,625  (12,947,222 to 13,133,217) | 163.26  (161.41 to 165.05) | 79,859  (78,438 to 81,362) | 59% | 21,188,727 | 16.72 |
| Arexvy and Abrysvo | 218 | 12,633,949  (12,546,899 to 12,719,611) | 158.58  (156.89 to 160.30) | 79,669  (78,273 to 81,052) | 68% | 20,850,529 | 16.45 |

**
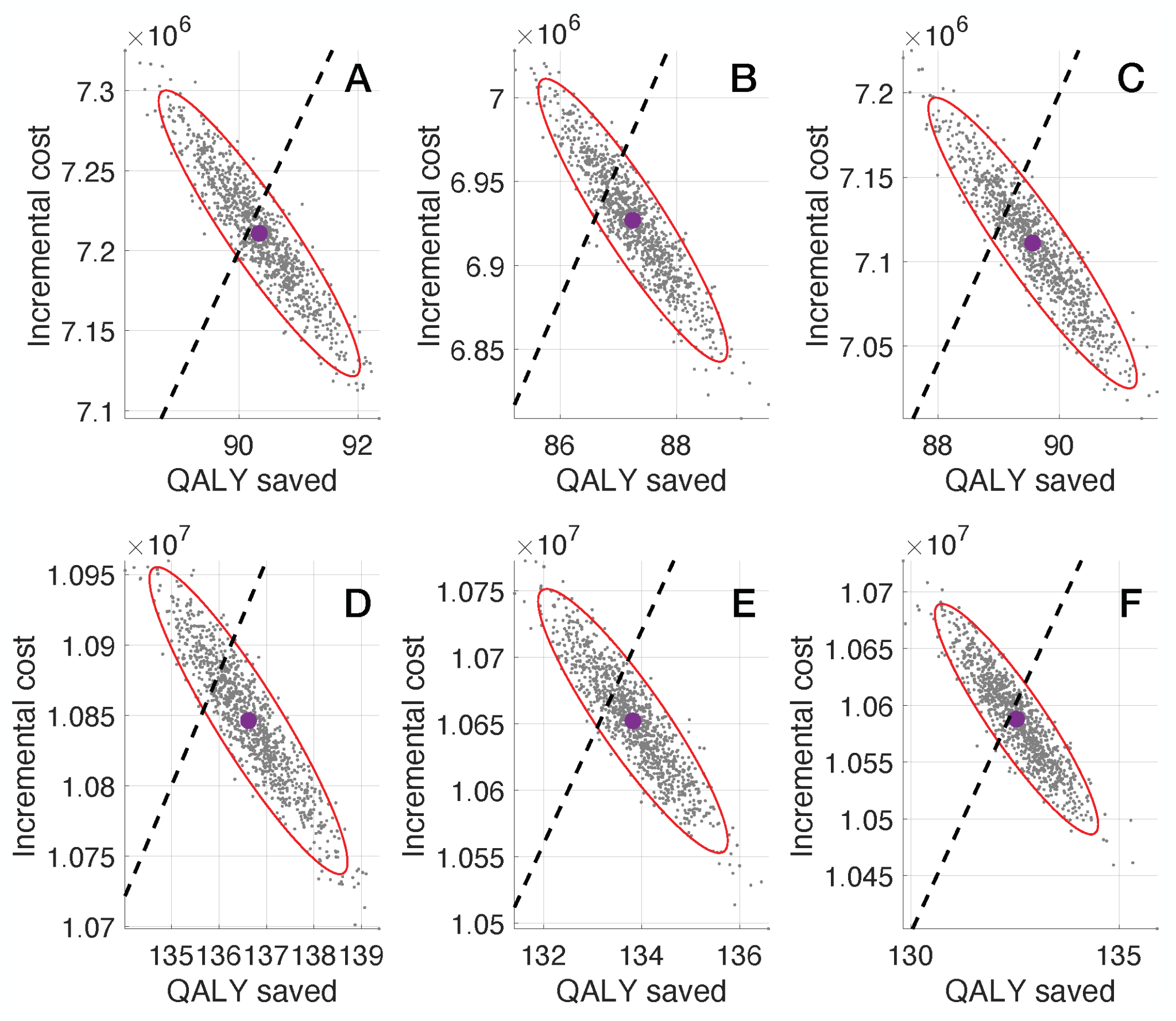
Figure A22.** Cost-effectiveness planes for vaccination programs over two RSV seasons with sigmoidal vaccine efficacy profiles under S1 (A,B,C) and S2 (D,E,F). Scenarios correspond to: (A) Arexvy alone with PPD of $190; (B) Abrysvo alone with PPD of $177; a combination of Arexvy and Abrysvo with PPD of $184; (D) Arexvy alone with PPD of $189; (B) Abrysvo alone with PPD of $179; and a combination of Arexvy and Abrysvo with PPD of $181. Black dashed-line corresponds to the WTP threshold of $80,000. Red curve presents the associated 95% credible ellipse of the data points distribution.


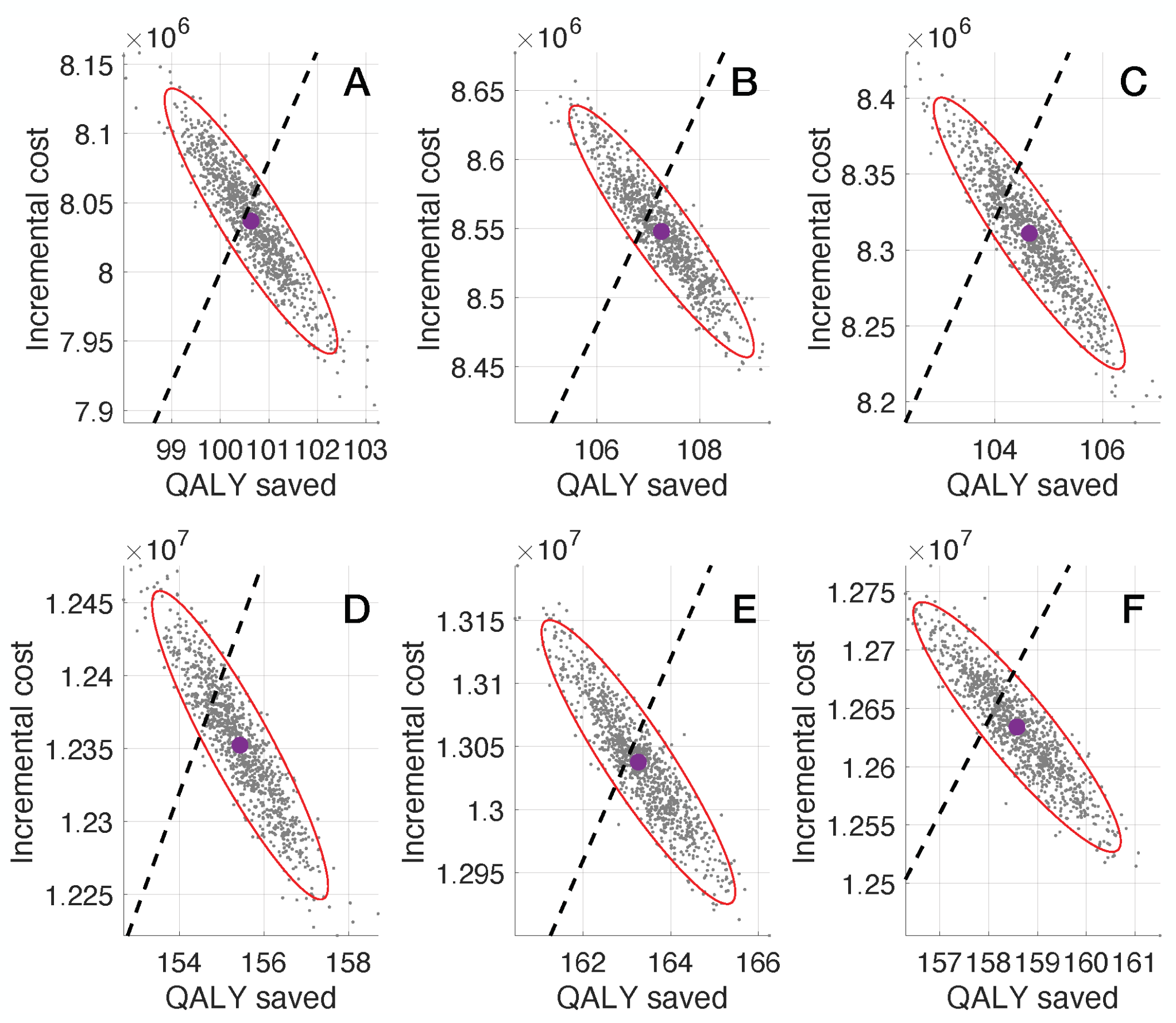
**Figure A23.** Cost-effectiveness planes for vaccination programs over two RSV seasons with sigmoidal vaccine efficacy profiles under S1 (A,B,C) and S2 (D,E,F). Scenarios correspond to: (A) Arexvy alone with PPD of $212; (B) Abrysvo alone with PPD of $221; a combination of Arexvy and Abrysvo with PPD of $217; (D) Arexvy alone with PPD of $215; (B) Abrysvo alone with PPD of $222; and a combination of Arexvy and Abrysvo with PPD of $218. Black dashed-line corresponds to the WTP threshold of $80,000. Red curve presents the associated 95% credible ellipse of the data points distribution.

**Cost-effectiveness analysis with the WTP of $120,000 per QALY gained during the first RSV season**

**
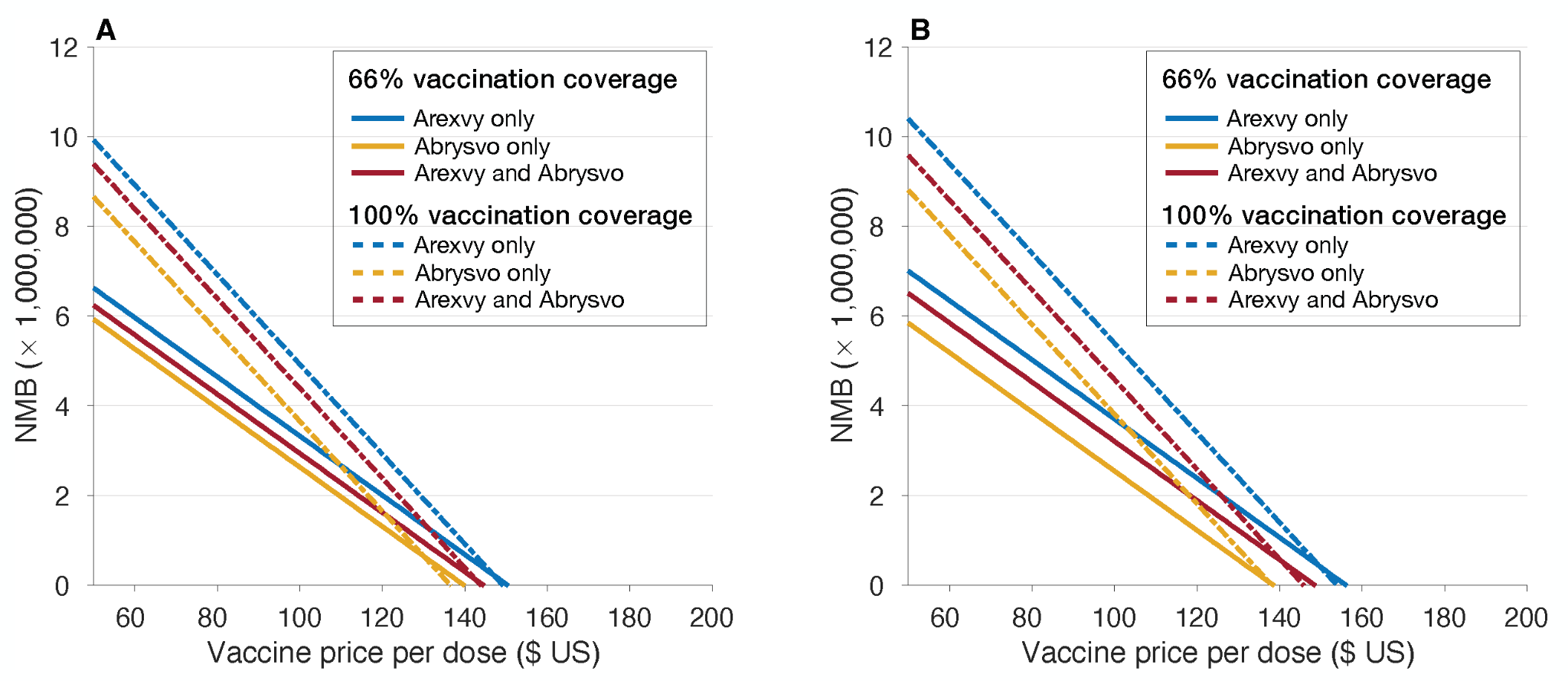
Figure A24.** Estimated net monetary benefit (NMB) during the first RSV season as a function of price per dose for Arexvy and Abrysvo with different coverage of vaccination, and sigmoidal (A) and linear (B) vaccine efficacy profiles. For scenarios using both Arexvy and Abrysvo, each vaccine was assumed to have 50% of the target coverage with the same price per dose.

**Table A13.** Model estimates of cost-effectiveness analyses for vaccination programs with Arexvy only, Abrysvo only, and combination of Arexvy and Abrysvo during the first RSV season in a population of 100,000 adults aged 60 years or older at the WTP of $120,000. All strategies were compared to the baseline with no intervention.

| **Scenario** | **Maximum**  **PPD, $** | **Incremental costs, $**  **(95% CI)** | **QALY saved**  **(95% CI)** | **ICER**  **(95% CI)** | **Probability of being cost-effective** | **Budget impact per 100,000, $** | **National budget impact, $ billion** |
| --- | --- | --- | --- | --- | --- | --- | --- |
| *S1 with sigmoidal vaccine efficacy* | | | | | | | |
| Arexvy only | 150 | 7,080,363  (7,028,302 to 7,132,552) | 59.19  (58.25 to 60.14) | 119,629  (116,865 to 122,434) | 61% | 10,217,485 | 8.06 |
| Abrysvo only | 139 | 6,754,121  (6,707,784 to 6,802,761) | 56.71  (55.82 57.67) | 119,089  (116,281 to 121,750) | 74% | 9,595,939 | 7.57 |
| Arexvy and Abrysvo | 144 | 6,892,866  (6,840,861 to 6,943,542) | 57.74  (56.78 to 58.72) | 119,382  (116,590 to 122,181) | 67% | 9,881,527 | 7.80 |
| *S2 with sigmoidal vaccine efficacy* | | | | | | | |
| Arexvy only | 149 | 10,668,448  (10,614,923 to 10,724,116) | 89.06  (88.09 to 90.09) | 119,794  (117,845 to 121,696) | 58% | 15,392,291 | 12.15 |
| Abrysvo only | 136 | 10,036,899  (9,976,114 to 10,095,439) | 84.01  (82.87 to 85.10) | 119,467  (117,274 to 121,743) | 67% | 14,249,858 | 12.45 |
| Arexvy and Abrysvo | 143 | 10,390,977  (10,332,919 to 10,448,981) | 87.27  (86.23 to 88.31) | 119,067  (117,077 to 121,115) | 83% | 14,879,360 | 11.74 |
| *S1 with linear vaccine efficacy* | | | | | | | |
| Arexvy only | 156 | 7,355,240  (7,302,678 to 7,405,915) | 61.37  (60.45 to 62.31) | 119,844  (117,135 to 122,512) | 55% | 10,598,880 | 8.36 |
| Abrysvo only | 138 | 6,686,596  (6,638,638 to 6,735,812) | 55.99  (55.06 to 56.87) | 119,416  (116,743 to 122,331) | 66% | 9,523,178 | 7.52 |
| Arexvy and Abrysvo | 148 | 7,067,828  (7,015,478 to 7,124,281) | 59.21  (58.21 to 60.15) | 119,372  (116,703 to 122,335) | 68% | 10,132,022 | 8.00 |
| *S2 with linear vaccine efficacy* | | | | | | | |
| Arexvy only | 153 | 10,927,058  (10,871,284 to 10,985,392) | 91.80  (90.77 to 92.78) | 119,033  (117,189 to 121,001) | 85% | 15,761,485 | 12.44 |
| Abrysvo only | 138 | 10,140,563  (10,080,804 to 10,197,330) | 84.56  (83.48 to 85.55) | 119,924  (117,882 to 122,088) | 53% | 14,432,195 | 11.39 |
| Arexvy and Abrysvo | 145 | 10,490,016  (10,434,388 to 10,545,572) | 88.12  (87.15 to 89.10) | 119,048  (117,117 to 121,005) | 83% | 15,042,803 | 11.87 |

**
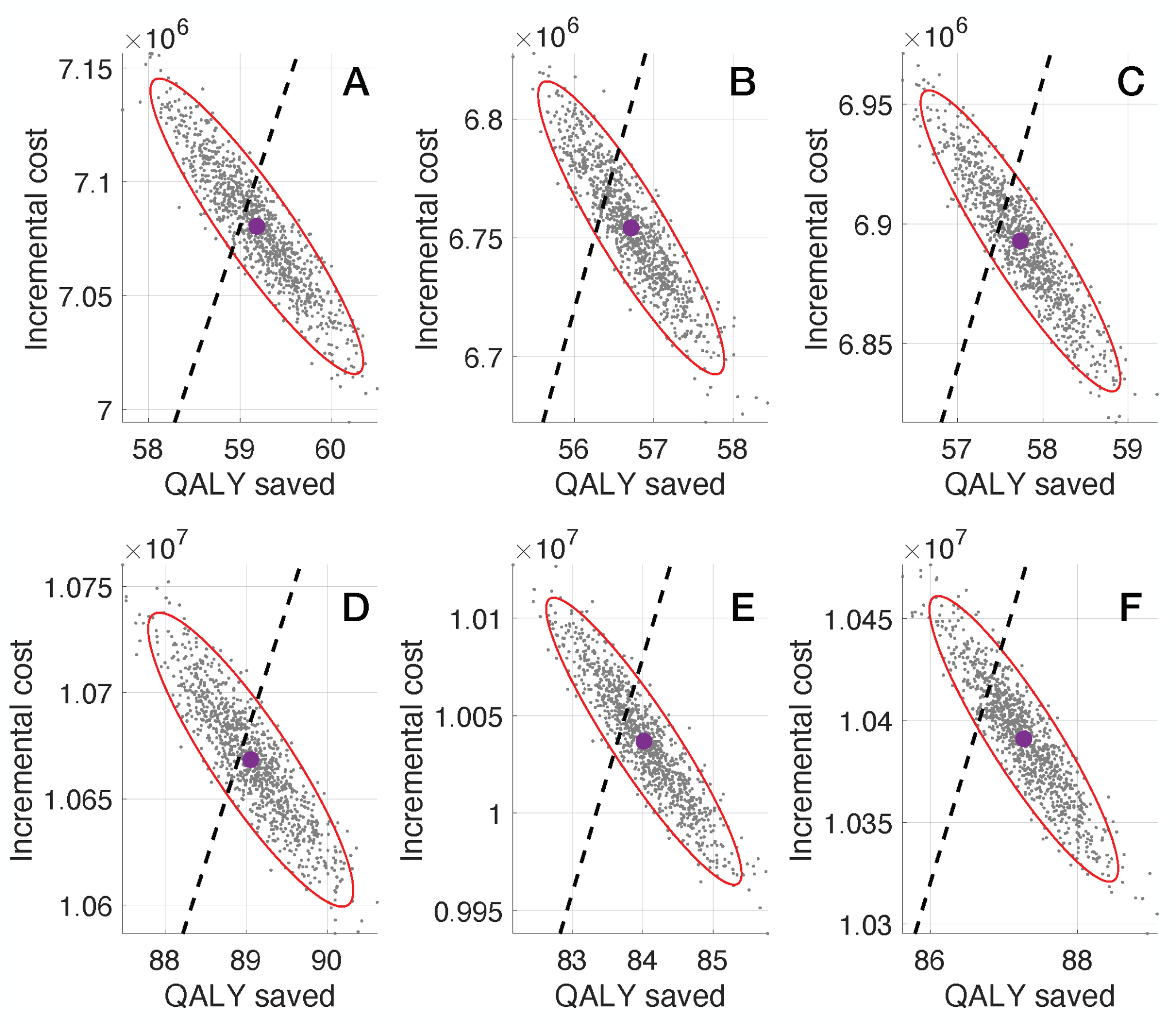
Figure A25.** Cost-effectiveness planes for vaccination programs during the first RSV season with sigmoidal vaccine efficacy profiles under S1 (A,B,C) and S2 (D,E,F). Scenarios correspond to: (A) Arexvy alone with PPD of $150; (B) Abrysvo alone with PPD of $139; a combination of Arexvy and Abrysvo with PPD of $144; (D) Arexvy alone with PPD of $149; (B) Abrysvo alone with PPD of $136; and a combination of Arexvy and Abrysvo with PPD of $143. Black dashed-line corresponds to the WTP threshold of $120,000. Red curve presents the associated 95% credible ellipse of the data points distribution.

**
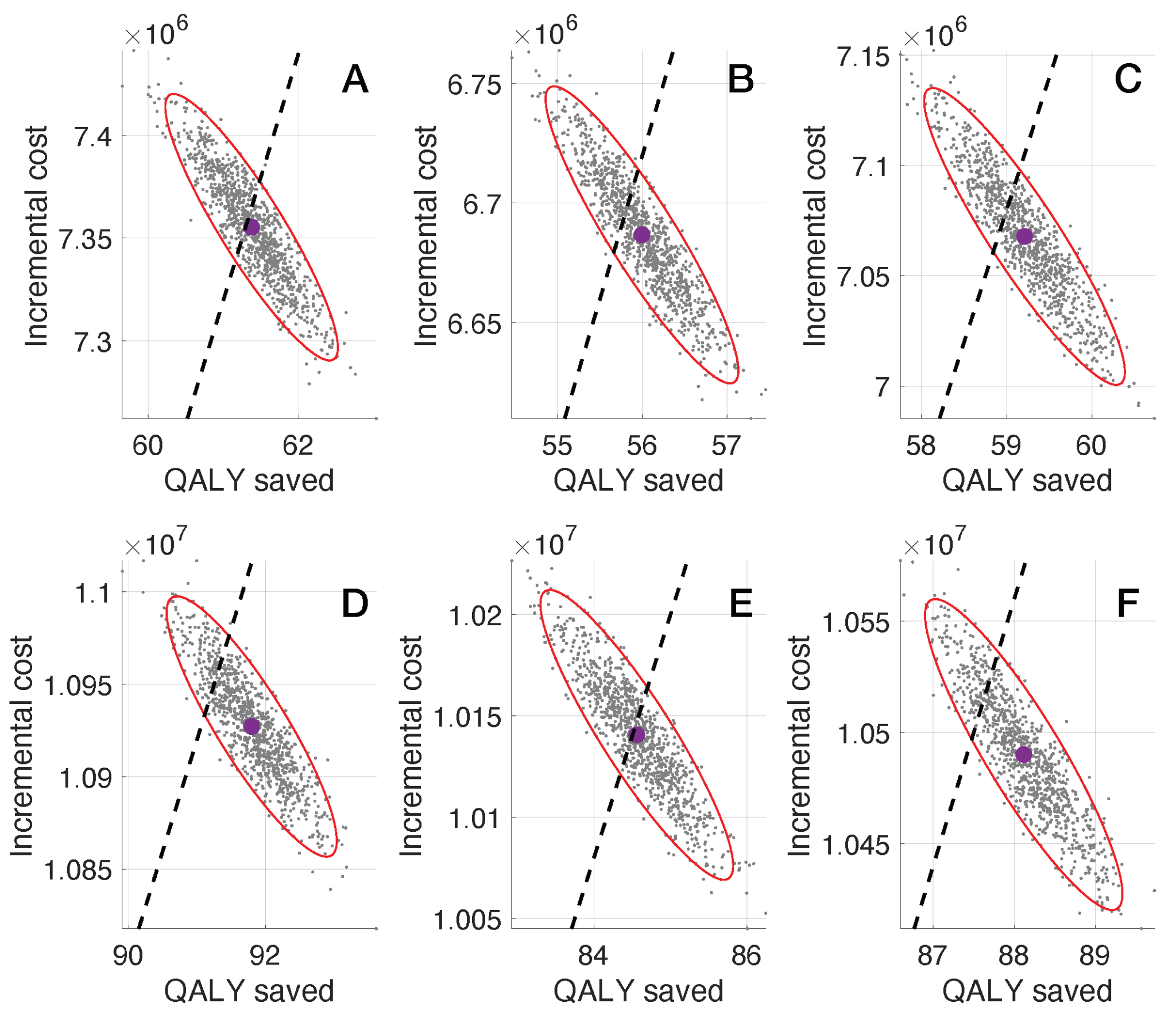
Figure A26.** Cost-effectiveness planes for vaccination programs during the first RSV season with linear vaccine efficacy profiles under S1 (A,B,C) and S2 (D,E,F). Scenarios correspond to: (A) Arexvy alone with PPD of $156; (B) Abrysvo alone with PPD of $138; a combination of Arexvy and Abrysvo with PPD of $148; (D) Arexvy alone with PPD of $153; (B) Abrysvo alone with PPD of $138; and a combination of Arexvy and Abrysvo with PPD of $145. Black dashed-line corresponds to the WTP threshold of $120,000. Red curve presents the associated 95% credible ellipse of the data points distribution.

**Cost-effectiveness analysis with the WTP of $12,000 per QALY gained over two RSV seasons**

**
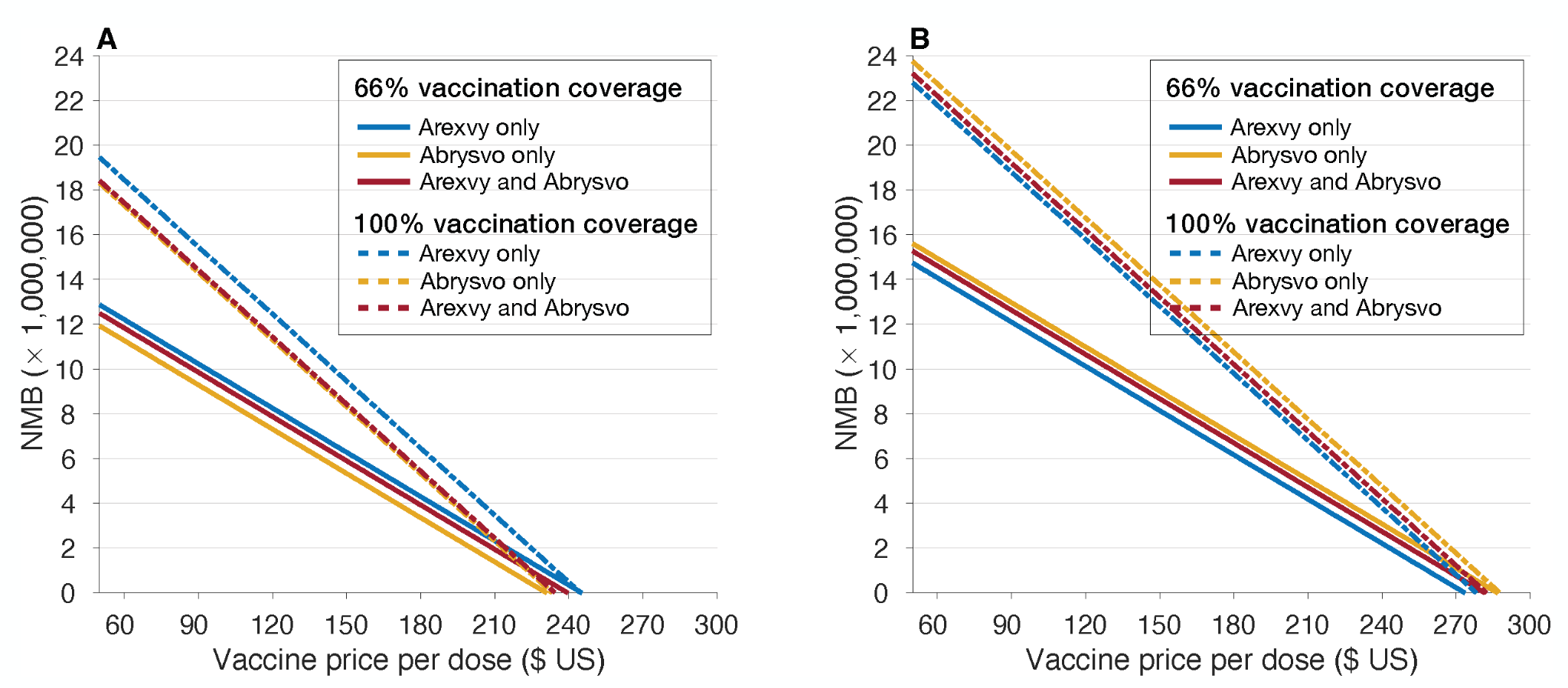
Figure A27.** Estimated net monetary benefit (NMB) over two RSV seasons as a function of price per dose for Arexvy and Abrysvo with different coverage of vaccination, and sigmoidal (A) and linear (B) vaccine efficacy profiles. For scenarios using both Arexvy and Abrysvo, each vaccine was assumed to have 50% of the target coverage with the same price per dose.

**Table A14.** Model estimates of cost-effectiveness analyses for vaccination programs with Arexvy only, Abrysvo only, and combination of Arexvy and Abrysvo over two RSV seasons in a population of 100,000 adults aged 60 years or older at the WTP of $120,000. All strategies were compared to the baseline with no intervention.

| **Scenario** | **Maximum**  **PPD, $** | **Incremental costs, $**  **(95% CI)** | **QALY saved**  **(95% CI)** | **ICER**  **(95% CI)** | **Probability of being cost-effective** | **Budget impact per 100,000, $** | **National budget impact, $ billion** |
| --- | --- | --- | --- | --- | --- | --- | --- |
| *S1 with sigmoidal vaccine efficacy* | | | | | | | |
| Arexvy only | 244 | 10,774,775  (10,701,609 to 10,847,687) | 90.34  (88.95 to 91.68) | 119,272  (116,686 to 121,905) | 72% | 15,701,126 | 12.39 |
| Abrysvo only | 230 | 10,424,983  (10,356,851 to 10,493,116) | 87.25  (85.96 to 88.58) | 119,485  (116,972 to 122,015) | 66% | 14,871,137 | 11.74 |
| Arexvy and Abrysvo | 239 | 10,740,981  (10,673,066 to 10,810,003) | 89.56  (88.18 to 90.88) | 119,936  (117,519 to 122,590) | 51% | 15,424,646 | 12.17 |
| *S2 with sigmoidal vaccine efficacy* | | | | | | | |
| Arexvy only | 244 | 16,346,207  (16,253,092 to 16,429,062) | 136.62  (135.11 to 138.38) | 119,647  (117,499 to 121,597) | 62% | 23,790,119 | 18.77 |
| Abrysvo only | 233 | 16,052,045  (15,975,988 to 16,129,776) | 133.82  (132.30 to 135.33) | 119,954  (118,122 to 121,876) | 52% | 22,836,450 | 18.02 |
| Arexvy and Abrysvo | 234 | 15,887,845  (15,810,078 to 15,970,292) | 132.54  (131.00 to 133.99) | 119,877  (118,018 to 121,869) | 55% | 22,866,018 | 18.04 |
| *S1 with linear vaccine efficacy* | | | | | | | |
| Arexvy only | 273 | 12,062,741  (11,979,770 to 12,133,600) | 100.63  (99.27 to 102.16) | 119,871  (117,364 to 122,147) | 53% | 17,430,770 | 13.76 |
| Abrysvo only | 286 | 12,837,852  (12,766,772 to 12,915,639) | 107.25  (105.82 to 108.59) | 119,695  (117,576 to 122,029) | 61% | 18,223,549 | 14.38 |
| Arexvy and Abrysvo | 281 | 12,534,996  (12,461,395 to 12,605,363) | 104.64  (103.18 to 106.03) | 119,795  (117,596 to 122,156) | 56% | 17,928,999 | 14.15 |
| *S2 with linear vaccine efficacy* | | | | | | | |
| Arexvy only | 278 | 18,652,398  (18,568,805 to 18,735,653) | 155.43  (153.76 to 157.05) | 120,004  (118,279 to 121,828) | 50% | 26,906,286 | 21.23 |
| Abrysvo only | 287 | 19,537,625  (19,447,222 to 19,633,217) | 163.26  (161.41 to 165.05) | 119,673  (117,801 to 121,617) | 65% | 27,688,727 | 21.85 |
| Arexvy and Abrysvo | 282 | 19,033,949  (18,946,899 to 19,119,611) | 158.58  (156.89 to 160.30) | 120,027  (118,166 to 121,848) | 49% | 27,250,529 | 21.50 |

**
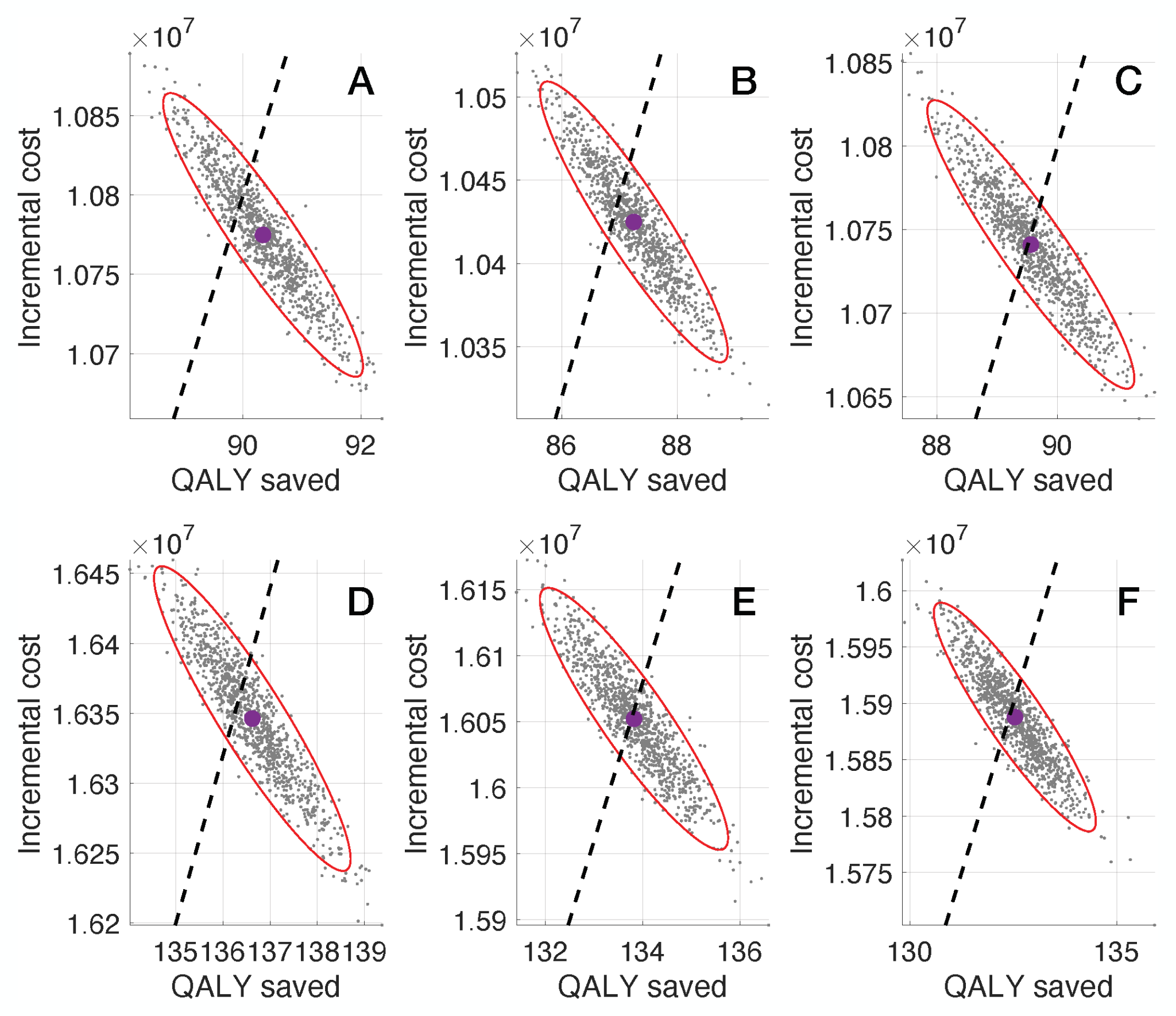
Figure A28.** Cost-effectiveness planes for vaccination programs over two RSV seasons with sigmoidal vaccine efficacy profiles under S1 (A,B,C) and S2 (D,E,F). Scenarios correspond to: (A) Arexvy alone with PPD of $244; (B) Abrysvo alone with PPD of $230; a combination of Arexvy and Abrysvo with PPD of $239; (D) Arexvy alone with PPD of $244; (B) Abrysvo alone with PPD of $233; and a combination of Arexvy and Abrysvo with PPD of $234. Black dashed-line corresponds to the WTP threshold of $120,000. Red curve presents the associated 95% credible ellipse of the data points distribution.

**
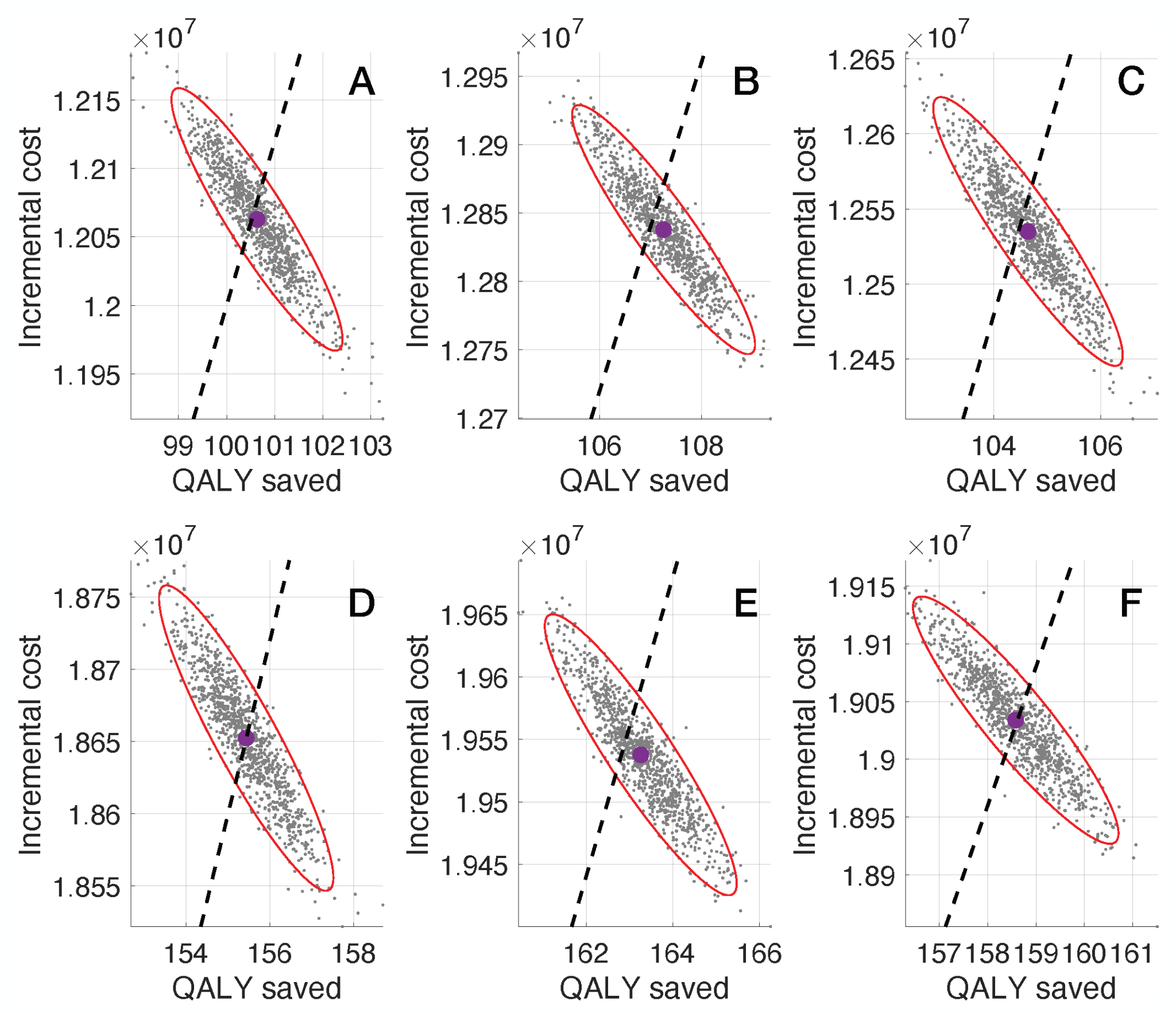
Figure A29.** Cost-effectiveness planes for vaccination programs over two RSV seasons with sigmoidal vaccine efficacy profiles under S1 (A,B,C) and S2 (D,E,F). Scenarios correspond to: (A) Arexvy alone with PPD of $273; (B) Abrysvo alone with PPD of $286; a combination of Arexvy and Abrysvo with PPD of $281; (D) Arexvy alone with PPD of $278; (B) Abrysvo alone with PPD of $287; and a combination of Arexvy and Abrysvo with PPD of $282. Black dashed-line corresponds to the WTP threshold of $120,000. Red curve presents the associated 95% credible ellipse of the data points distribution.

**References**

[1. Piccirillo, J. F. *et al.* The Changing Prevalence of Comorbidity Across the Age Spectrum. *Crit. Rev. Oncol. Hematol.* **67**, 124–132 (2008).](https://www.zotero.org/google-docs/?KURwJV)

[2. Erickson, P., Wilson, R. & Shannon, I. Years of healthy life. *Healthy People 2000 Stat. Notes* 1–15 (1995) doi:10.1037/e583992012-001.](https://www.zotero.org/google-docs/?KURwJV)

[3. US Centers for Disease Control and Prevention. RSV-NET Interactive Dashboard. https://www.cdc.gov/rsv/research/rsv-net/dashboard.html (2023).](https://www.zotero.org/google-docs/?KURwJV)

[4. US Bureau of Labor Statistics. Civilian labor force, by age, sex, race, and ethnicity : U.S. Bureau of Labor Statistics. https://www.bls.gov/emp/tables/civilian-labor-force-summary.htm.](https://www.zotero.org/google-docs/?KURwJV)

[5. Grosse, S. D., Krueger, K. V. & Pike, J. Estimated annual and lifetime labor productivity in the United States, 2016: implications for economic evaluations. *J. Med. Econ.* **22**, 501–508 (2019).](https://www.zotero.org/google-docs/?KURwJV)

[6. Papi, A. *et al.* Respiratory Syncytial Virus Prefusion F Protein Vaccine in Older Adults. *N. Engl. J. Med.* **388**, 595–608 (2023).](https://www.zotero.org/google-docs/?KURwJV)

[7. Walsh, E. E. *et al.* Efficacy and Safety of a Bivalent RSV Prefusion F Vaccine in Older Adults. *N. Engl. J. Med.* **388**, 1465–1477 (2023).](https://www.zotero.org/google-docs/?KURwJV)

[8. Gessner, B. D. The cost-effectiveness of a hypothetical respiratory syncytial virus vaccine in the elderly. *Vaccine* **18**, 1485–1494 (2000).](https://www.zotero.org/google-docs/?KURwJV)
